# A CRISPR-Cas9-based reporter system for single-cell detection of extracellular vesicle-mediated functional transfer of RNA

**DOI:** 10.1038/s41467-020-14977-8

**Published:** 2020-02-28

**Authors:** Olivier G. de Jong, Daniel E. Murphy, Imre Mäger, Eduard Willms, Antonio Garcia-Guerra, Jerney J. Gitz-Francois, Juliet Lefferts, Dhanu Gupta, Sander C. Steenbeek, Jacco van Rheenen, Samir El Andaloussi, Raymond M. Schiffelers, Matthew J. A. Wood, Pieter Vader

**Affiliations:** 1 Laboratory of Clinical Chemistry and Hematology, University Medical Center Utrecht, Utrecht, The Netherlands; 2 Department of Paediatrics, University of Oxford, Oxford, United Kingdom; 3 Department of Physiology, Anatomy and Genetics, University of Oxford, Oxford, United Kingdom; 4 Department of Physics, University of Oxford, Oxford, United Kingdom; 5 Pediatric Pulmonology and Regenerative Medicine Center, University Medical Center Utrecht, Utrecht, The Netherlands; 6 Department of Laboratory Medicine, Clinical Research Center, Karolinska Institutet, Huddinge, Sweden; 7 Department of Molecular Pathology, Oncode Institute, Netherlands Cancer Institute, Amsterdam, The Netherlands; 8 Department of Experimental Cardiology, University Medical Center Utrecht, Utrecht, The Netherlands

**Keywords:** Nanoparticles, Assay systems, Membrane trafficking

## Abstract

Extracellular vesicles (EVs) form an endogenous transport system for intercellular transfer of biological cargo, including RNA, that plays a pivotal role in physiological and pathological processes. Unfortunately, whereas biological effects of EV-mediated RNA transfer are abundantly studied, regulatory pathways and mechanisms remain poorly defined due to a lack of suitable readout systems. Here, we describe a highly-sensitive CRISPR-Cas9-based reporter system that allows direct functional study of EV-mediated transfer of small non-coding RNA molecules at single-cell resolution. Using this CRISPR operated stoplight system for functional intercellular RNA exchange (CROSS-FIRE) we uncover various genes involved in EV subtype biogenesis that play a regulatory role in RNA transfer. Moreover we identify multiple genes involved in endocytosis and intracellular membrane trafficking that strongly regulate EV-mediated functional RNA delivery. Altogether, this approach allows the elucidation of regulatory mechanisms in EV-mediated RNA transfer at the level of EV biogenesis, endocytosis, intracellular trafficking, and RNA delivery.

## Abstract (web summary)

Extracellular vesicles (EV) facilitate intercellular transfer of biological material including RNA, but the regulatory mechanisms for their formation and transfer are incompletely known. Here the authors develop a CRISPR-based reporting system to detect the transfer of guide RNAs and identify genes not previously linked to EV-mediated RNA delivery.

## Introduction

Extracellular vesicles (EVs) are a heterogeneous population of small lipid membrane vesicles^1^, which play a role in intercellular communication through the transfer of biological macromolecules, consisting of both soluble and (trans)membrane proteins, lipids, and RNA molecules^2–5^. EVs are conventionally classified into two major subtypes based on their biogenesis: exosomes and microvesicles^1,5^. Exosomes are formed in the endosomal pathway, when intraluminal vesicle formation in the late endosome results in the formation of multivesicular bodies (MVBs)^1,6^. These MVBs may fuse with the plasma membrane resulting in the release of the intraluminal vesicles, upon which these vesicles are termed exosomes. Alternatively, microvesicles are released directly from the plasma membrane. Together these EV populations form an endogenous transport system through which numerous molecules, including various RNA species, are transferred between cells^5^. Over the last decade it has become clear that EV-mediated RNA transfer plays a critical role in the regulation of numerous physiological and pathological processes including immunomodulation, angiogenesis, cell proliferation, neurodegenerative pathologies, cardiovascular events, and tumor metastasis^4,7–11^. This has resulted in a vast increase in studies on EVs in the context of cell biology, homeostasis, novel targets for therapeutic intervention in pathologies, as well as a potential new source for biomarkers in diagnostics. Moreover, due to their endogenous capability of RNA transfer, EVs have sparked major interest as a potential therapeutic strategy for drug delivery, as well as regenerative medicine applications^12^.

Despite the high number of studies focusing on EV-mediated RNA transfer in health and disease, fundamental studies on RNA uptake and processing mechanisms are currently lacking, mainly due to the absence of suitable readout systems to study RNA transfer^13,14^. Current studies on EV uptake generally measure the uptake of fluorescently labelled EVs, either by use of fluorescent (membrane) dyes or by use of fluorescently labelled proteins that are enriched in EVs^12,15^. Such studies have provided valuable information regarding general EV uptake mechanisms and dynamics, but are not necessarily representative of EV cargo delivery. To address these issues, reporter systems based on mRNA transfer, such as the Cre-LoxP reporter system, have been employed^8,16^. However, EV-mediated transfer of large mRNA molecules such as Cre recombinase mRNA, a molecule of >350 kDa (over 1000 nt, excluding post-transcriptional modifications) may differ from loading, transfer, and processing of small RNAs. This is underlined by multiple studies that have shown that EVs from various sources contain mainly small (~100 nt) RNA molecules and only trace amounts of full length mRNA^17–23^, and that the majority of mRNA in EVs is not present as intact mRNAs^24,25^. Moreover, a major drawback of mRNA-based systems is that it is inherently impossible to phenotypically distinguish between reporter activation as a result of the delivery of translated protein or the mRNA itself^26^, which reduces the applicability of such systems to study RNA transfer specifically.

To overcome these challenges, we aimed to design a novel approach to study functional RNA delivery, capable of activating high expression of a fluorescent reporter protein, independent of translation of the RNA molecule. To this end, we explored the suitability of the CRISPR/Cas9 genome editing system. The CRISPR/Cas9 system is a gene‐editing technique where the Cas9 protein is guided to a specific genomic sequence by a ~100 nucleotide, 35 kD, single guide RNA (sgRNA), resulting in a specific double‐stranded break in the genomic DNA^27^. Due to inaccuracies in the non-homologous end joining (NHEJ) repair mechanism, frameshifts may occur around the targeted genomic region^28^. sgRNA molecules are highly suitable candidates to study functional intercellular RNA exchange, as the functionality of sgRNAs in this system is not based on RNA translation but rather on its secondary structure.

To visualize the transfer of sgRNAs, we designed a fluorescent “Stoplight” reporter system which is permanently activated in EV-acceptor cells upon functional transfer of a specific targeting sgRNA, expressed in EV-donor cells. Using this approach, we demonstrate functional intercellular sgRNA transfer using direct co-culture, transwell, and direct EV-addition experiments at single-cell resolution. Moreover, we establish protocols to study the effects of siRNA-mediated knockdown (KD) of single targets in both EV-acceptor and donor cells, as well as inhibitory compounds, on EV-mediated functional RNA transfer. First, we confirm the suitability of this system to study the role of specific genetic targets that we and other have previously shown to be pivotal for EV endocytosis and intracellular membrane trafficking. Then, using these protocols, we uncover several novel genes involved in the regulation of specific EV subtype biogenesis, as well as endocytosis and intracellular membrane trafficking that play a regulatory role in EV-mediated functional RNA delivery.

Altogether, we demonstrate a CRISPR/Cas9-based reporter system that allows the study of functional delivery of small non-coding RNAs with single-cell resolution. This novel approach allows the study of EV cargo processing in the context of functional RNA delivery, and may help to increase our understanding of the regulatory pathways that dictate the underlying processes. We term this approach the CRISPR Operated Stoplight System for Functional Intercellular RNA Exchange (CROSS-FIRE).

## Results

### Generation of a fluorescent CRISPR/Cas9 Stoplight reporter

**Fig. 1:**
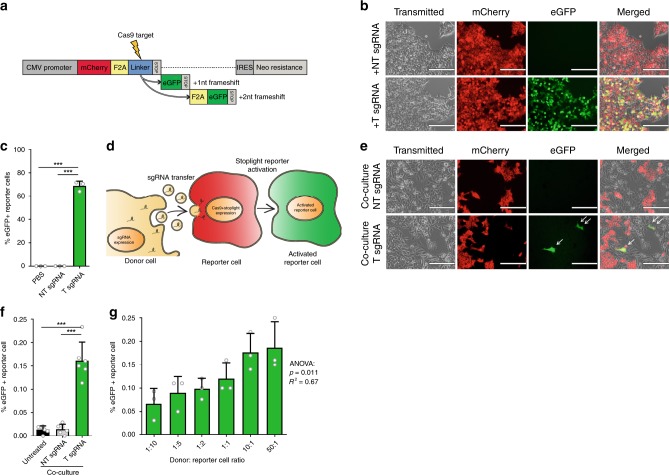
Establishment of a CRISPR/Cas9-activated fluorescence reporter platform to study EV-mediated RNA transfer. **a** Schematic showing the CRISPR/Cas9-activated fluorescent stoplight reporter system. mCherry is expressed under a CMV promoter, followed by a Cas9-targeted linker region and a stop codon. Two eGFP open reading frames are placed after the stop codon, one or two nucleotides (nt) out of frame, respectively. Upon a Cas9-mediated frameshift in the linker region, either one of these eGFP open reading frames will be permanently expressed alongside mCherry. F2A self-cleaving peptide domains are placed between each fluorescent protein. **b** Fluorescent microscopy images of stable HEK293T Stoplight^+^spCas9^+^ cells after transfection of a plasmid encoding a sgRNA targeting the linker region of the Stoplight construct (+T sgRNA, bottom row), or a non-targeting sgRNA (+NT sgRNA, top row). Scale bar represents 200 µm. Representative images as observed in three independent experiments. **c** Flow cytometry analysis of stable HEK293T Stoplight^+^spCas9^+^ cells after addition of PBS, transfection of a non-targeting sgRNA (NT sgRNA), or a sgRNA targeting the Stoplight construct (T sgRNA). Means + SD, *n* = 3 independent experiments, Student’s *t*-test. **d** Cartoon explaining the CROSS-FIRE system. Donor cells (yellow cell, left) express sgRNAs targeting a stoplight construct, which is expressed alongside Cas9 in reporter cells (red cell, middle). Upon functional transfer of sgRNAs from the donor cells to the reporter cell, Cas9 and sgRNA will together activate the stoplight construct in the reporter cell, resulting in permanent eGFP expression (green cell, right), which may then be quantified by fluorescence microscopy or flow cytometry. **e**, **f** A five day co-culture of HEK293T Stoplight^+^spCas9^+^ reporter cells with MDA-MB-231 sgRNA^+^ donor cells expressing a targeting sgRNA (T sgRNA), or a non-targeting sgRNA (NT sgRNA), analyzed by fluorescence microscopy (**e**) and flow cytometry (**f**). Scale bar represents 200 µm. Representative images as observed in six independent experiments Means + SD, *n* = 6 independent experiments, Tukey’s multiple comparison test. **g** Quantification of a five day co-culture of HEK293T Stoplight^+^spCas9^+^ reporter cells with MDA-MB-231 sgRNA^+^ donor cells in varying donor cell: reporter cell ratios by flow cytometry. Means + SD, *n* = 3 independent experiments, ANOVA. ***p <0.001.

To evaluate the intercellular transfer of RNAs, we designed a fluorescent “Stoplight” reporter system that constitutively expresses mCherry, followed by a “linker” region between mCherry and its stop codon which can be targeted by Cas9. Upon sgRNA delivery and subsequent NHEJ-mediated frameshift generation in this linker region of either +1 nt or +2 nt, the original stop codon will be bypassed, eliciting a permanently expressed eGFP signal (Fig. 1a). First, the Stoplight reporter construct was stably incorporated into HEK293T cells to confirm its functionality. As expected, Stoplight^+^ HEK293T cells showed high expression of mCherry, but only showed eGFP expression after transfection of both *Streptococcus pyogenes* Cas9 (spCas9) and a targeting sgRNA (Supplementary Fig. 1A). In order to generate a reporter exclusively for sgRNA delivery/transfer, stable Stoplight^+^spCas9^+^ HEK293T cells were generated, and subsequently transfected with plasmids encoding either a targeting sgRNA (T sgRNA), or a non-targeting sgRNA (NT sgRNA) control. As confirmed by fluorescence microscopy (Fig. 1b), flow cytometry (Fig. 1c, Supplementary Fig. 2), and in silico image-based analysis of confocal microscopy images (Supplementary Fig. 3A–C), Stoplight^+^spCas9^+^ cells expressing T sgRNA showed high levels of eGFP expression, whereas reporter cells expressing NT sgRNA, or left untreated, did not. Observed levels of activation of eGFP expression were in line with in Delphi in silico indel and frameshift predictions (Supplementary Fig. 1B, C) which, based on the target sequence, predicted a frameshift frequency of +1 nt or +2 nt of approx. 80%^29^.

### Intercellular transfer of sgRNAs

Having validated the Stoplight reporter construct, we assessed whether “donor” cells expressing sgRNAs were capable of activating the Stoplight reporter system via transfer of sgRNAs to “reporter” cells (illustrated in Fig. 1d), an approach which we term as CRISPR operated stoplight system for functional intercellular RNA exchange (CROSS-FIRE). To this end, stable sgRNA^+^ MDA-MB-231 donor lines were generated, expressing either T sgRNAs or NT sgRNAs, and co-cultured with a Stoplight^+^spCas9^+^ HEK293T reporter line. Co-culture of reporter cells with T sgRNA expressing donor cells resulted in significant reporter activation within five days, whereas co-culture with donor cells expressing NT sgRNAs did not (Fig. 1e–f and Supplementary Fig. 3D). Moreover, employing different donor:reporter cell ratios demonstrated reporter activation in a dose-dependent manner (Fig. 1g). Overall, the percentages of reporter activation after five days were found to be low (up to 0.2%). However, the observed low percentages of reporter activation do not necessarily reflect a low level of EV-mediated communication, but rather are the result of the low levels of sgRNA in EVs as we opted not to employ additional strategies for targeted loading of EVs with sgRNAs, such as RNA-binding proteins fused to EV-associated proteins, in order to study RNA loading and transfer in an unbiased manner.

To confirm that these observations were not due to reporter cell-line specific characteristics we generated five additional stable Stoplight^+^spCas9^+^ reporter cell lines using HeLa, HMEC-1, MCF-7, MDA-MB-231, and T47D cells. Similar to HEK293T reporter cells, all five cell lines showed a dose-dependent Stoplight reporter activation after co-culture with sgRNA^+^ MDA-MB-231 donor cells (Supplementary Fig. 4). Concordantly, various additional sgRNA^+^ donor cell lines commonly used for functional EV studies were generated: HEK293T, HMEC-1, and hTERT-MSC cells. Interestingly, co-culture of HEK293T Stoplight^+^spCas9^+^ reporter cells with sgRNA^+^ HMEC-1 and hTERT-MSC resulted in significant reporter activation within five days, whereas co-culture with sgRNA^+^ HEK293T did not (Supplementary Fig. 5).

Having demonstrated functional sgRNA transfer between multiple cell types in a co-culture setting, we deemed it important to rule out sgRNA transfer via cell-cell fusion. Therefore, we generated Gaussia luciferase (G.Luc)^+^sgRNA^+^ donor cells, which were co-cultured with Stoplight^+^spCas9^+^ reporter cells. After co-culture, cells were separated based on eGFP and mCherry expression by fluorescence activated cell sorting (FACS), and after recovery were subjected to a luciferase activity assay (as illustrated in Supplementary Fig. 6A). In case of cell-cell fusion, eGFP^+^ reporter cells should also show luciferase activity. After having confirmed luciferase activity in the stable sgRNA^+^G.Luc^+^ donor cells (Supplementary Fig. 6B), a seven day co-culture was performed and mCherry^−^eGFP^−^ (donor cells), mCherry^+^eGFP^−^ (unactivated reporter cells), and mCherry^+^eGFP^+^ (activated reporter cells) cells were isolated, as confirmed by fluorescence microscopy (Supplementary Fig. 6C). A luciferase assay on conditioned medium of these cell populations showed strong luciferase activity in the mCherry^−^eGFP^−^ donor cells, but no activity in untreated medium and conditioned medium from both mCherry^+^eGFP^−^ and mCherry^+^eGFP^+^ cells (Supplementary Fig. 6D), excluding transfer of sgRNAs via cell-cell fusion. We were also unable to detect luciferase activity in sgRNA^+^G.Luc^+^ donor cell-derived EVs (Supplementary Fig. 6E, F).

**Fig. 2:**
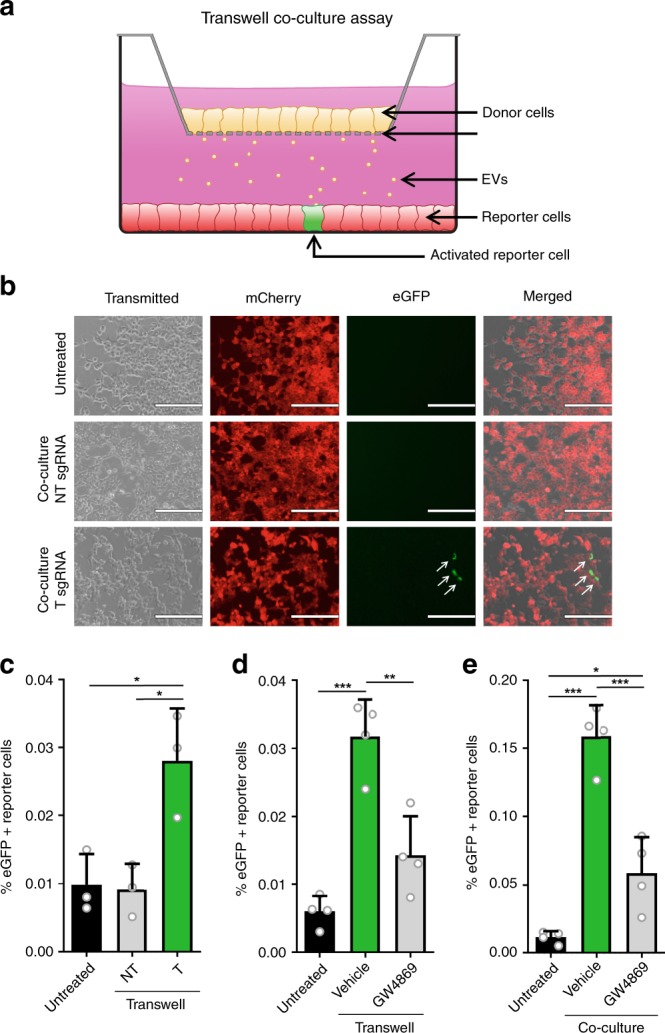
Cell contact is not required for intercellular sgRNA exchange. **a** A schematic cartoon explaining the use of a transwell co-culture assay using the CROSS-FIRE system. sgRNA^+^ donor cells are cultured in a transwell insert (yellow cells, top), which is suspended in a standard tissue culture well containing Stoplight^+^spCas9^+^ reporter cells, allowing for exchange of secreted factors while avoiding direct cell contact. **b**, **c** Fluorescence microscopy pictures (**b**) and flow cytometry analysis (**c**) of HEK293T Stoplight^+^spCas9^+^ reporter cells after a 10-day transwell co-culture experiment with MDA-MB-231 sgRNA^+^ donor cells expressing a non-targeting (NT) or a targeting (T) sgRNA. Scale bar represents 200 µm. Means + SD, *n* = 3 independent experiments, Tukey’s multiple comparison test. **d** Flow cytometry analysis of HEK293T Stoplight^+^spCas9^+^ reporter cells after a 10-day transwell co-culture experiment with MDA-MB-231 sgRNA^+^ donor cells expressing a targeting (T) sgRNA with or without the presence of EV release inhibitor GW4869 at a concentration of 1 μM. Means + SD, *n* = 4 independent experiments, Tukey’s multiple comparison test. **e** Flow cytometry analysis of HEK293T Stoplight^+^spCas9^+^ reporter cells after a five day direct co-culture experiment with MDA-MB-231 sgRNA^+^ donor cells expressing a targeting (T) sgRNA with or without the presence of EV release inhibitor GW4869 at a concentration of 1 μM. Means + SD, *n* = 4 biologically independent samples, Tukey’s multiple comparison test. *p < 0.05, **p < 0.01, ***p < 0.001.

To investigate cell-contact independent transfer of sgRNA, we tested the CROSS-FIRE system in a transwell co-culture assay (Fig. 2a). In concordance with previous observations, co-culture with T sgRNA^+^ resulted in significant reporter activation, whereas transwell co-culture with NT sgRNA^+^ donor cells did not (Fig. 2b, c), demonstrating that direct cell-cell contact is not required for sgRNA transfer. In this assay, we observed a significant but notably lower level of activation as compared to direct co-culture protocols. We hypothesize that this difference is due to the lower number of donor cells as a result of the small transwell membrane surface, as well as limited availability (and potential blockage) of pores that facilitate EV transfer, as also seen in other studies^8^. As an extension of this finding, another transwell co-culture experiment was performed in the presence of GW4869, an nSMAse inhibitor which strongly inhibits EV release^30^. Indeed, the addition of GW4869 resulted in a strong and significant decrease in reporter activation (Fig. 2d). Similarly, presence of GW4869 also resulted in a substantial and significant decrease of reporter activation in a direct co-culture experiment as confirmed by flow cytometry (Fig. 2e) and in silico image-based analysis of confocal microscopy images (Supplementary Fig. 3E). Altogether, these data suggest that functional sgRNA transfer is mediated by EVs.

### EV-mediated transfer of sgRNAs

**Fig. 3:**
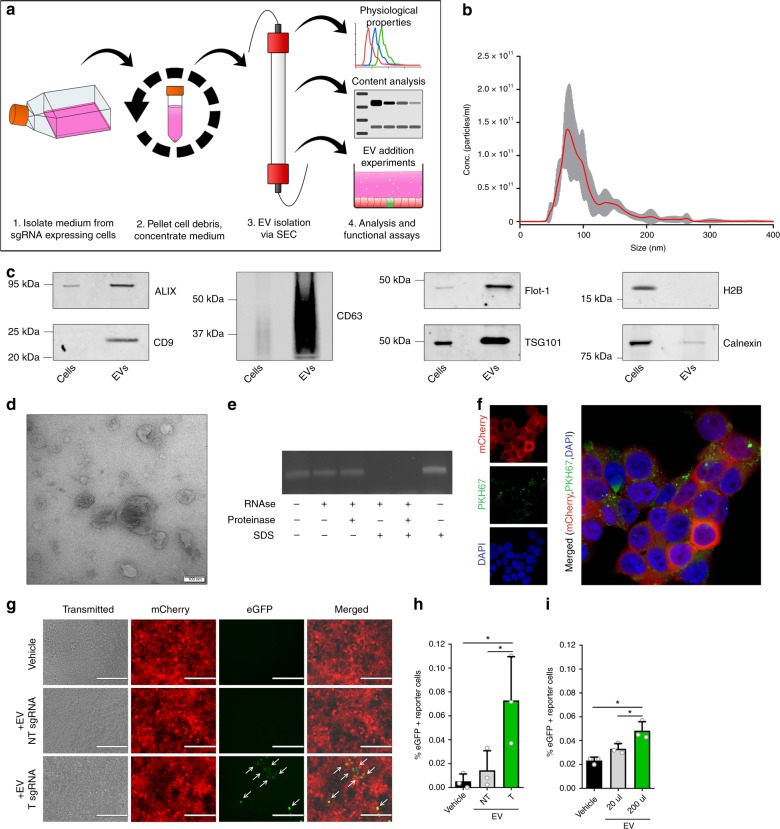
Intercellular sgRNA exchange is EV-mediated. **a** Schematic of EV isolation workflow and analysis. Conditioned medium is isolated from sgRNA^+^ MDA-MB-231 donor cells, after which cell debris is pelleted through centrifugation. Conditioned medium is then concentrated by tangential flow filtration, followed by size exclusion chromatography-mediated EV isolation. EVs are then characterized, and used for EV addition experiments in Stoplight^+^spCas9^+^ reporter cells. **b** Nanosight Nanoparticle Tracking Analysis (NTA) showing the size distribution of the isolated EVs (mode = 81 ± 3 nm). **c** Western blot analysis of EVs and cell lysates for common EV markers (CD9, CD63, ALIX, Flot-1, and TSG101), and EV-negative markers Calnexin and H2B. Representative images as observed in three independent experiments. **d** Electron microscopy analysis of isolated EVs. Scale bar represents 100 nm. Representative image as observed in 12 separate fields. **e** An EV RNase protection assay, followed by RT-PCR sgRNAs, shows that sgRNAs are only susceptible to RNase-mediated degradation in the presence of the membrane-disrupting anionic surfactant SDS. Representative data as observed in three independent experiments. **f** Confocal microscopy images of HEK293T reporter cells (red) that have taken up MDA-MB-231-derived PKH67-labeled EVs (green). Representative image as observed in seven randomly selected fields. **g**, **h** EV-mediated activation of the CROSS-FIRE platform using EVs isolated from sgRNA^+^ MDA-MB-231 donor cells. EVs were added every 72 h for 12 additions with an average dose of 1.1e11 ± 4.9e10 EVs per addition. Cells were analyzed by fluorescence microscopy (**g**) and flow cytometry (**h**). Scale bar represents 200 µm. Means + SD, *n* = 3 biological replicates, Tukey’s multiple comparison test. **i** EVs from sgRNA^+^ MDA-MB-231 donor cells activate Stoplight^+^ spCas9^+^ HEK293T reporter cells in a dose-dependent manner. EVs were added every 72 h for nine additions with an average concentration of 2.2e10 ± 1e10 (20 µl) or 2.2e11 ± 1e11 (200 µl). Means + SD, *n* = 3 biological replicates, Tukey’s multiple comparison test. *p < 0.05.

To confirm this hypothesis, we tested the direct functionality of isolated EVs in the CROSS-FIRE system. EVs were isolated from sgRNA^+^ MDA-MB-231 donor cell conditioned medium through size exclusion chromatography (SEC) (Fig. 3a and Supplementary Fig. 7A). Isolated EVs were characterized according to the Minimal Information for Studies of Extracellular Vesicles (MISEV) guidelines^31^. Nanoparticle Tracking Analysis (NTA) showed a size-distribution profile in line with EV characteristics (Fig. 3b) (mode = 81 ± 3 nm), and Western Blot analysis showed an enrichment for common EV-markers ALIX, Flot-1, TSG101, and tetraspanins CD9 and CD63, alongside a strong decrease in abundance of nuclear marker histone H2B and organelle marker Calnexin (Fig. 3c). Furthermore, transmission electron microscopy (TEM) of isolated EVs showed lipid bi-layer vesicles displaying common EV morphology, as well as a size-distribution in line with NTA measurements (Fig. 3d). The presence of sgRNA in EVs was confirmed by RT-PCR, and an EV RNase protection assay revealed that sgRNAs were present in the lumen of these EVs, as RNase-mediated degradation of sgRNAs was only observed after SDS-mediated membrane disruption (Fig. 3e). Using a synthetic sgRNA standard curve, we determined an abundance of 1 sgRNA per approx. 4.5e5 EVs by qPCR on RNA isolated from sgRNA^+^ MDA-MB-231 donor cells in combination with NTA analysis (Supplementary Fig. 7B, C). To confirm that these isolated EVs were able to be taken up by reporter cells, isolated EVs were fluorescently labelled with PKH67 lipid-dye, and administered to Stoplight^+^ HEK293T cells, followed by confocal microscopy analysis. This experiment revealed that labelled EVs were indeed readily taken up by reporter cells (Fig. 3f). Using isolated EVs, we confirmed significant activation of CROSS-FIRE reporter cells by EV addition when isolating EVs from T sgRNA^+^ donor cells, but not from NT sgRNA^+^ donor cells, as determined by both fluorescence microscopy (Fig. 3g) and flow cytometry (Fig. 3h). EV-mediated reporter activation was also confirmed by in silico image-based analysis of confocal microscopy images (Supplementary Fig. 3F). Moreover, EVs dose-dependently activated reporter cells (Fig. 3i). EV-mediated CROSS-FIRE reporter activation was not affected by RNase A treatment of the EVs (Supplementary Fig. 7D). Additionally, we show that addition of the soluble protein-containing fractions isolated alongside these EVs by size exclusion chromatography did not result in the activation of the reporter cells (Supplementary Fig. 7E). These data show that the CROSS-FIRE reporter system is activated by EV-mediated sgRNA transfer.

### Single gene analysis in intercellular RNA transfer

**Fig. 4:**
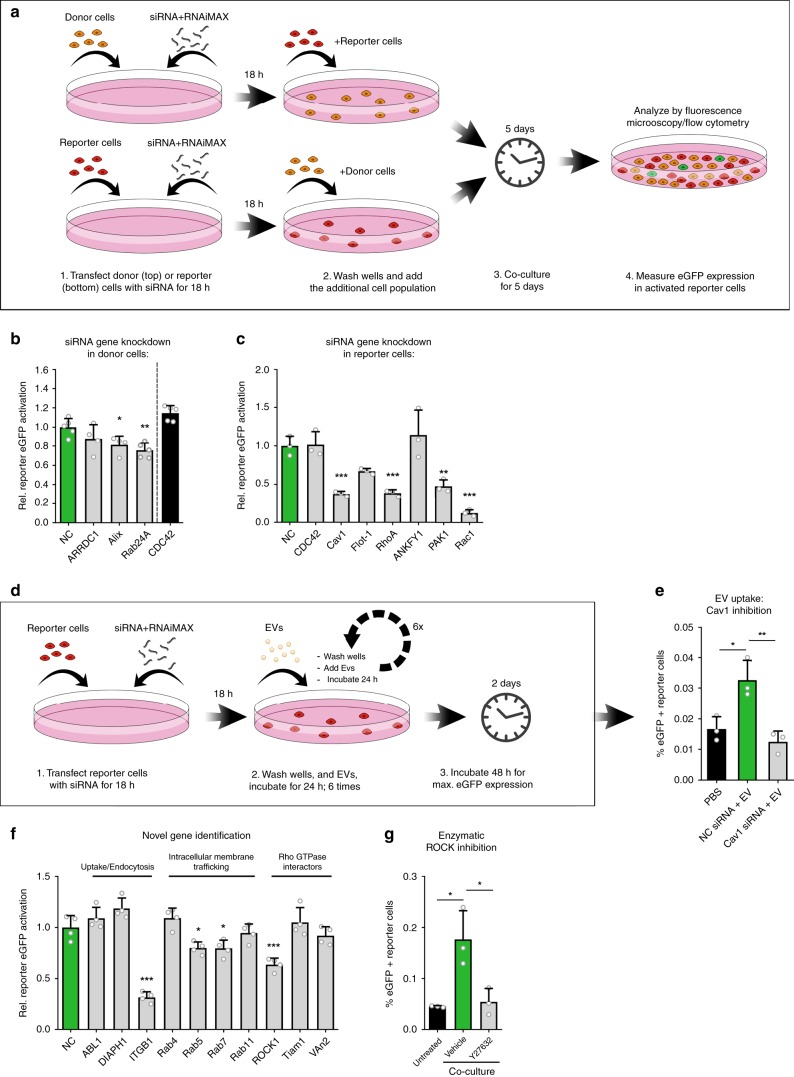
Analysis of cellular pathways to study EV-mediated RNA transfer and uptake. **a** Workflow to study the role of specific genetic targets in intercellular RNA transfer using the CROSS-FIRE system. **b** Flow cytometry analysis of HEK293T Stoplight^+^spCas9^+^ reporter cells after a five day co-culture with sgRNA^+^ MDA-MB-231 donor cells subjected to siRNA-mediated KD of genes as compared to a non-targeting negative control siRNA (NC). Means + SD, *n* = 5 independent experiments, Dunnett’s multiple comparison test. **c** Flow cytometry analysis of HEK293T Stoplight^+^spCas9^+^ reporter cells after a five day co-culture with sgRNA^+^ MDA-MB-231 donor cells, in which HEK293T reporter cells were subjected to siRNA-mediated KD of various genes as compared to a non-coding control siRNA (NC). Means + SD, *n* = 4 independent experiments, Dunnett’s multiple comparison test. **d** Schematic of workflow to study the role of genes involved in EV-mediated RNA delivery. **e** Flow cytometry analysis of Stoplight^+^spCas9^+^ HEK293T reporter cells treated for six consecutive days with sgRNA^+^ MDA-MB-231-derived EVs after transfection with a non-coding siRNA (NC), or siRNAs targeting Cav1, as compared to vehicle-treated reporter cells (PBS). EVs were added every 24 h for six additions with an average concentration of 1.8e11 ± 1.1e11 EVs per addition. Means + SD, *n* = 3 biological replicates, Tukey’s multiple comparison test. **f** Flow cytometry analysis of HEK293T Stoplight^+^spCas9^+^ reporter cells after a five day co-culture with sgRNA^+^ MDA-MB-231 donor cells, in which HEK293T reporter cells were subjected to siRNA-mediated KD of various novel genes as compared to a non-coding control siRNA (NC). Means + SD, *n* = 4 independent experiments, Dunnett’s multiple comparison test. **g** Flow cytometry analysis of HEK293T reporter cells after a five day direct co-culture experiment with sgRNA^+^MDA-MB-231 donor cells with or without the presence of ROCK-inhibitor Y27632 at a concentration of 1 μM. Means + SD, *n* = 3 biologically independent samples, Tukey’s multiple comparison test. *p < 0.05, **p < 0.01, ***p < 0.001.

We next established a CROSS-FIRE based workflow to study specific target genes and pathways in EV-mediated RNA transfer by donor or reporter cell exclusive siRNA-mediated target knockdown (KD), as illustrated in Fig. 4a. As a proof of concept, the effect of knocking down various genes with known involvements in EV biogenesis in MDA-MB-231 donor cells was evaluated (Fig. 4b). Whereas the KD of ARRDC1 (involved in release of a subpopulation of microvesicles) showed no significant effect on RNA transfer, KD of Alix and Rab27A (involved in exosome biogenesis and release, respectively) resulted in a significant decrease of reporter activation, as compared to a non-targeting siRNA negative control (NC)^32,33^. Recently a potentially novel process for intercellular RNA transfer, exchange through tunneling nanotubes, was described, in which CDC42 plays a pivotal regulatory role^34^. Interestingly, siRNA-mediated KD of CDC42 in our system showed no effect on intercellular functional RNA exchange. These experiments, alongside a direct co-culture experiment in the presence of EV release inhibitor GW4869 were repeated using Stoplight^+^spCas9^+^ MCF-7 reporter cells, yielding similar results: as observed with HEK293T reporter cells, sgRNA transfer was significantly decreased in the presence of GW4869 (Supplementary Fig. 8A) and by KD of Alix and Rab27A, but not ARRDC1 and CDC42, in donor cells (Supplementary Fig. 8B). These data demonstrate that the CROSS-FIRE system is capable and suitable to study the role of different EV subpopulations in functional RNA transfer.

We then employed this CROSS-FIRE based workflow to study EV-mediated RNA delivery and processing, by targeting various regulatory genes of endocytosis and intracellular membrane trafficking in HEK293T recipient reporter cells. Using this method, we uncovered various genes that are pivotal for EV-mediated RNA transfer: Rho GTPases Rac1 and RhoA, the Rho GTPase effector PAK1, and Cav1, involved in endocytosis (Fig. 4c). KD of Rho GTPase CDC42, as well as ANKFY1 (involved in intracellular vesicle transport) and Flot-1 (involved in endocytosis), showed no effect on RNA transfer. In alignment with HEK293T cells, KD of Cav1, Rac1 and RhoA in MCF-7 reporter cells also resulted in significant inhibition of reporter activation, and KD of CDC42 and Flot-1 did not result in significant differences. Interestingly, KD of ANKFY1 and PAK1 in MCF-7 had a different effect than in HEK293T cells: whereas PAK1 KD in MCF-7 cells did not result in a significant decrease in reporter activation, KD of ANKFY1 resulted in a 1.9-fold increase in reporter activation (Supplementary Fig. 8C). These findings underline the importance of confirming such pathways in multiple cell lines, as previous studies have shown that relative roles of various endocytic routes may vary between different cell types^12,35,36^. Moreover, these findings demonstrate that with the CROSS-FIRE system it is now possible to study the separate roles of relevant pathways in EV-mediated RNA delivery. As a proof of concept, we tested the suitability of the CROSS-FIRE system to study the contribution of specific genes to the RNA delivery process using isolated EVs. To this end, Cav1 was knocked down in reporter cells, which were subsequently treated with six doses of sgRNA^+^ EVs, and analyzed by flow cytometry (as illustrated in Fig. 4d). In line with previous observations, reporter cells stimulated with sgRNA^+^ EVs showed significant reporter activation whereas reporter cells treated with a Cav1-targeting siRNA did not (Fig. 4e).

Based on these results, we selected ten new genetic targets involved in endocytosis (ABL1, DIAPH1), extracellular matrix adhesion (ITGB1), intracellular membrane trafficking (Rab4, Rab5, Rab7, and Rab11), and Rho GTPase interactors (RhoA effector ROCK1, and Rac1 interactors Tiam1 and VAV2) to study their role in functional RNA delivery to recipient reporter cells (Fig. 4f). Of these ten genetic targets, four targets that were not yet previously linked to EV-mediated RNA delivery appeared to play an important role in functional RNA delivery: integrin ITGB1 (also known as CD29), Rab5 and Rab7 (important for early endosome and late endosome trafficking, respectively), and ROCK1 (downstream target protein kinase of RhoA). To further confirm the role of ROCK1 in RNA transfer, the effect of ROCK1 inhibitor Y27632 in a direct five days co-culture experiment was assessed (Fig. 4g). Indeed, addition of 1 µM Y27632 substantially and significantly decreased reporter activation. Altogether, these data show that the CROSS-FIRE system provides a robust and scalable approach to study and uncover novel regulatory targets and pathways in intercellular RNA exchange in a direct co-culture setting, or using isolated EVs.

### Pol II-mediated sgRNA expression

**Fig. 5:**
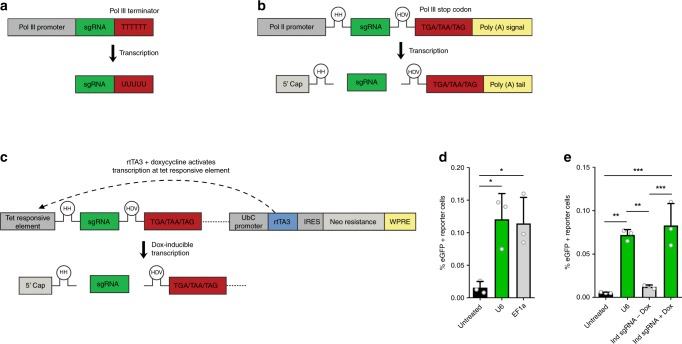
Incorporation of self-cleaving RNA ribozymes allows the use of Pol II-mediated donor cell sgRNA expression in the CROSS-FIRE system. **a** Standard expression of sgRNAs under a Pol III promoter. Transcription is ended by a Pol III terminator sequence; TTTTTT. The expressed sgRNA is not subjected to any additional post-transcriptional modifications under a Pol III promoter. **b** Incorporation of self-cleaving Hammerhead (HH) and Hepatitis Delta Virus (HDV) RNA ribozymes sequences flanking the sgRNA sequence expressed under a Pol II promoter results in the removal of post-transcriptional Pol II modifications. **c** A construct for doxycycline-inducible expression of sgRNAs using a Pol II TET-On inducible system, by incorporation of self-cleaving RNA ribozymes. **d** Flow cytometry analysis of Stoplight^+^spCas9^+^ HEK293T reporter cells after a five day co-culture with sgRNA^+^ MDA-MB-231 donor cells expressing sgRNAs under a U6 Pol III promoter (**a**), or a EF1a Pol II promoter using self-cleaving RNA ribozymes (**b**). Means + SD, *n* = 3 independent experiments, Tukey’s multiple comparison test. **e** Flow cytometry analysis of Stoplight^+^spCas9^+^ HEK293T reporter cells after a five day co-culture with sgRNA^+^ MDA-MB-231 donor cells expressing sgRNAs under regulation of a U6 Pol III promoter (**a**), and a doxycycline-inducible promoter Pol II promoter (**c**). Means + SD, *n* = 3 biologically independent samples, Tukey’s multiple comparison test. *p < 0.05, **p < 0.01, ***p < 0.001.

In closing, we modified sgRNA expression constructs to allow for a more adaptable design of CROSS-FIRE based studies. In all experiments described above, sgRNAs were expressed under a Pol III U6 promoter. This is a common strategy for sgRNA expression^27,28^, as it allows ubiquitous high expression, with minimal post-transcriptional modifications (Fig. 5a)^37^. However, Pol III promoters provide limited options for transcriptional regulation. In contrast, Pol II promoters allow for more versatile experimental designs for RNA expression^38^, but result in substantial post-transcriptional modifications that interfere with sgRNA functionality^39,40^. Recently, a novel technique for sgRNA multiplexing under a single promoter was described using self-cleaving ribozymes^41^. We employed this approach to remove Pol II-mediated RNA modifications, allowing Pol II-mediated unmodified sgRNA expression. As a proof of concept, this approach was tested for expression under an EF1a promoter (Fig. 5b), as well as in a doxycycline (dox)-inducible Tet-ON system (Fig. 5c)^42^. Indeed, CROSS-FIRE co-culture experiments confirmed the functionality of both systems (Fig. 5d, e), without a decrease of efficiency as compared to the Pol III U6 promoter. This modification further expands the potential of the CROSS-FIRE system, allowing future use of inducible or tissue-specific regulation of sgRNA expression in donor cells.

## Discussion

Studies over the last decade have shown that there is a natural transport system, extracellular vesicles (EV), which allows cells to transfer proteins, mRNA and microRNA (miRNA) to other cells^3,43,44^, and by doing so play a role in numerous physiological and pathological processes^45^. EVs have become a topic of great interest as potential therapeutic targets in a variety of pathologies, as well as for designing novel therapeutic drug delivery strategies^5^. Functional EV-mediated transfer of RNA molecules relies on uptake of the target cells, as well as subsequent specific intracellular trafficking and processing. Even though recent studies have shown that EVs are capable of delivery of functional mRNA as well as miRNA to target cells^16,33^, a significant amount of EVs are transported to lysosomal compartments after uptake^15^. These findings indicate that EVs are suitable vectors for RNA delivery, but also underline that EV uptake and cargo delivery is a highly regulated and intricately complex process. Indeed, to date, much remains unknown about the mechanisms dictating EV targeting, internalization and intracellular trafficking, and the contributing EV components have not yet been characterized. Understanding the biology underlying the EV-based intercellular transfer of RNA is pivotal to gain a better understanding of the role of EVs in both physiological and pathophysiological processes, as potential therapeutic targets, as well as for a more rational development of EVs as drug delivery vehicles or EV-inspired synthetic systems.

In this manuscript, we describe a CRISPR/Cas9-based approach to study EV-mediated functional RNA transfer. This CROSS-FIRE is, to the best of our knowledge, the first system that allows the measurement of small non-coding RNA transfer at single-cell resolution. Such a system is pivotal to unravel the underlying mechanisms of EV-mediated RNA delivery, as currently employed methods either do not demonstrate functional content delivery (e.g., fluorescent dyes), or do not differentiate between RNA and protein delivery (mRNA-based reporter systems)^12–14,26^. Moreover, sensitivity of measuring EV-mediated transfer of miRNAs on whole cell populations is generally low, as small effects are masked by expression levels in the total cell population. The CROSS-FIRE system addresses all these issues, as reporter activation is based on functional delivery of sgRNAs, which do not rely on protein translation for their functionality^27^. Furthermore, as the reporter system read-out is high induction of a fluorescent signal, activation can be measured at single-cell resolution.

A previously designed fluorescent reporter system to study EV-mediated cargo transfer is the Cre-LoxP reporter system^8,16^. Like the CROSS-FIRE system, this reporter system is based on activation of a fluorescent protein in recipient reporter cells upon functional cargo delivery. However, rather than small RNA transfer, the Cre-LoxP reporter system is based on transfer of large Cre mRNA molecules or Cre protein. Therefore, we do not envision the CROSS-FIRE system as a replacement or competing tool of the Cre-LoxP system. Rather, we envision complementary roles for these reporter systems, in which the Cre-LoxP system may be utilized to study the transfer of larger mRNA molecules and Cre protein (as functional transfer of Cre protein currently cannot be ruled out), whereas the CROSS-FIRE system can be used to exclusively study the functional delivery of smaller RNA molecules. Indeed, sgRNAs appear to be a highly suitable RNA molecule to study EV-mediated RNA transfer, as multiple studies have shown that EVs contain higher levels of small RNAs around ~100 nt, whereas only traces amounts of full length mRNA have been detected^17–23^. Concordantly, it has been shown that the majority of mRNAs present in EVs are not present as intact mRNAs^24,25^. This could explain why we observed a higher sensitivity when comparing the CROSS-FIRE system to the Cre-LoxP reporter system in a five days co-culture experiment using the same donor and reporter cell combinations and ratios (Supplementary Fig. 9).

Despite the high sensitivity of the CROSS-FIRE reporter system, reporter activation in our experimental set-up was low (up to 0.2% in HEK293T cells). These data are not due to low levels of efficiency of EV-mediated communication, but rather the result of low amounts of sgRNA loaded into vesicles as no targeted RNA loading or enrichment strategies were employed. As a result, sgRNA abundance is approx. one RNA molecule per 4.5e5 EVs, an abundance which is not uncommon for naturally expressed RNA molecules^46,47^. Moreover, lower percentages of activation can likely be explained by the short time span of the experiments, as both co-culture and addition experiment protocols were designed for timeframes compatible with siRNA target KD protocols. Increasing the ratio of donor- to reporter cells and increasing the dose of sgRNA-containing EVs in addition experiments resulted in a dose-dependent increase of reporter activation, suggesting that indeed the amount of transferred sgRNA is a limiting factor in these experiments. Lastly, inDelphi in silico target sequence analysis predicted a frameshift frequency of approx. 80%, meaning that around 20% of all NHEJ-mediated mutations remain undetected^29^.

Interestingly, sgRNA transfer in co-culture experiments also appears to vary amongst different cell types. This appears to be the case for both reporter cells (Supplementary Fig. 4) and donor cells (Supplementary Fig. 5). These results are in line with observations from Zomer et al. using a Cre-LoxP-based reporter system to study EV-mediated cargo transfer from MDA-MB-231 donor cells to various reporter cell lines in co-culture experiments^8^. In line with our results, they observed a higher transfer of EV cargo from MDA-MB-231 cells to MCF-7 and T47D reporter cells than to MDA-MB-231 reporter cells (Supplementary Fig. 4). Moreover, we find that sgRNA transfer to reporter cells is also influenced by donor cell type. Whereas we see functional RNA transfer from MDA-MB-231 (epithelial breast cancer), HMEC-1 (microvascular endothelium), and hTERT-MSC (immortalized mesenchymal stem cell) donor cells within 5 days, no significant transfer was observed from HEK293T donor cells in these conditions (Supplementary Fig. 5). These data indicate that certain cell types may be less prone to exchange RNA with other cells. The latter observation is especially relevant to the field, as HEK293T cells are a commonly used cell source to study strategies for targeted RNA loading and delivery in EVs. It stands out that from these various donor cell lines, the highly metastatic MDA-MB-231 cells appear to show the highest functional transfer of sgRNAs to HEK293T reporter cells as compared to non-cancerous HEK293T, HMEC-1, and hTERT-MSC donor cells. These observations are in line with previous observations showing high levels of MDA-MB-231 EV-mediated intercellular communication with surrounding cells resulting in increased metastasis and tumor progression^8,48,49^. Based on these data it is thus tempting to conclude that tumor cells show higher levels of RNA transfer to other cells in general, as a result of increased EV secretion or more efficient uptake. However, based on these results alone we cannot definitively conclude that this applies to tumor cells in general.

In this manuscript, we optimized and demonstrate protocols that allow studying the role of single genetic targets in EV-mediated intercellular RNA transfer, by combining siRNA-mediated single target KD with both CROSS-FIRE co-culture and EV addition experiments. Such experiments are critical to unravel the underlying mechanisms that regulate EV-mediated RNA transfer, and to the best of our knowledge this is the first system that allows such experiments in a robust, scalable manner. Using this approach, we find that knocking down Alix and Rab27a (involved in EV production and release^32^) significantly decreases functional RNA transfer, whereas knocking down ARRDC1 (involved in microvesicle release^33^) and CDC42 (involved in tunneling nanotube regulation^34^) does not. These data show that the CROSS-FIRE system can be employed to assess the role of different EV subtypes in functional RNA transfer, a research topic that has recently gained substantial traction in the EV field. Such studies could greatly benefit the design of EV-based delivery systems by uncovering the most potent and suitable EV subpopulations for therapeutic RNA delivery. Additionally, such insights could lead to the discovery of novel ways of specific interfering with EV-mediated RNA transfer, a process involved in the communication between tumor cells and their surrounding tissues, affecting tumor growth and metastasis^8,48,49^.

Moreover, we studied the effect of knocking down various regulatory genes of endocytosis and intracellular membrane trafficking in reporter cells. We and others have previously observed that these pathways are involved in the regulation of EV uptake^12,35,36^. Indeed, in line with our previous observations on EV uptake, KD of Pak1 and Rac1, both involved in macropinocytosis, resulted in a significant decrease in reporter activation, whereas KD of ANKFY1 did not^12^. KD of Cav1 and RhoA involved in endocytosis, resulted in a substantial decrease of reporter activation, whereas targets CDC42 and Flot-1 showed no significant difference. The latter observation is of interest, as we previously demonstrated that Flot-1 KD does result in a significant decrease in EV uptake^12^. It is tempting to speculate that EVs taken up in a Flot-1-dependent manner could play a lesser role in EV-mediated RNA transfer, however additional experiments are required to fully elucidate this observation. Interestingly, knocking down macropinocytosis players PAK1 and ANKFY1 in MCF-7 reporter cells had virtually the opposite effect: whereas the inhibitory effect of PAK1 KD was absent in MCF-7 cells, knocking down ANKFY1 actually resulted in a substantial increase of functional RNA uptake by MCF-7 cells. These data demonstrate that the relative role of varying uptake routes may substantially differ between cell types, and showcases the complexity of such processes.

Lastly, we employed the CROSS-FIRE system to study the role of various novel genetic targets on EV-mediated RNA transfer (Fig. 4f). These experiments uncovered a role of four genes for functional sgRNA transfer: ITGB1 (integrin, extracellular matrix interaction), ROCK1 (downstream RhoA effector), and Rab5 and Rab7 (intracellular membrane trafficking). These findings confirm that the CROSS-FIRE system is suitable for uncovering novel genetic targets that are not only involved in EV uptake, but also in intracellular membrane trafficking. This once more underlines the importance of using read-outs that rely on functional RNA transfer to better understand their underlying mechanisms, in order to unravel the post-endocytotic processes that regulate EV-mediated RNA delivery. A better understanding of these mechanisms may greatly aid in the design of EV-mediated RNA-delivery strategies, as EV uptake and EV cargo processing in EV acceptor cells strongly dictate efficiency of RNA delivery^50^.

In summary, the CROSS-FIRE system is a highly sensitive method with broad applicability to study EV-mediated RNA delivery, and will help to increase our understanding of the regulatory pathways that dictate the underlying biological processes. This, in turn, holds the strong potential to provide a better understanding of the role of EV signaling in homeostasis and pathologies, and for uncovering, developing and implementing more efficient EV-mediated therapeutic strategies.

## Methods

### Cell culture

HEK293T, HeLa, MCF-7, and MDA-MB-231 cells were cultured in Dulbecco’s Modified Eagle Medium (DMEM) with l-Glutamine (Gibco) supplemented with 10% fetal bovine serum (FBS) (Sigma). T47D cells were cultured in DMEM/F12 medium with l-Glutamine supplemented with 10% FBS. hTERT-MSCs were cultured in alpha-MEM supplemented with GlutaMAX and 10% FBS. HMEC-1 cells were cultured in MCDB-131 medium supplemented with GlutaMAX, 10% FBS, 10 ng/ml rhEGF (Peprotech) and 50 nM Hydrocortisone (Sigma) on plates coated with 0.1% gelatin (Sigma). All cell lines were cultured in the presence of 100 µg/ml streptomycin, and 100 u/ml penicillin (Gibco) at 37 °C and 5% CO_2_.

### DNA constructs

The CROSS-FIRE fluorescent Stoplight reporter construct (Supplementary Table 1) was synthesized into a PG9-M2 vector by Gen9Bio. The fluorescent Stoplight reporter construct was subsequently cloned into a pHAGE2 lentiviral plasmid^51^; pHAGE2-CMV-IRES-NeoR, using the restriction enzymes NotI and BamHI (New England Biolabs) and a NEB Quick Ligation Kit (New England Biolabs). The fluorescent stoplight reporter construct was fully sequenced to rule out undesirable mutations. For stable spCas9 expression, a lentiCas9-P2A-Blast plasmid^52^ was used (Addgene #52962). For stable sgRNA expression, sgRNA targeting sequences were cloned into the lentiGuide-Puro plasmid^52^ (Addgene #52963), by ligating annealed complementary oligonucleotides into the plasmid after BsmBI digestion (New England Biolabs) using a NEB Quick Ligation Kit. Oligonucleotide sequences used for cloning are listed in Supplementary Table 2. For stable Cre Recombinase expression a pLV-CMV-Cre plasmid was used, and for stable expression of a fluorescent Cre Recombinase Stoplight reporter a pLV-CMV-LoxP-DsRed-LoxP-eGFP plasmid was used^8,16^. Oligonucleotides were synthesized by Integrated DNA Technologies. Pol II-compatible sgRNA sequences flanked by self-cleaving RNA ribozymes were designed as described by Gao et al.^41^ (Supplementary Table 3). Sequences were synthesized by Integrated DNA technologies, and cloned into pHAGE2-EF1a-UBC-PuroR or pInducer20^42^ plasmids using NotI and BamHI restriction enzymes, or BsmBI and AscI restricton enzymes, respectively.

### Lentiviral production and generation of stable cell lines

For lentiviral transduction of the CROSS-FIRE fluorescent Stoplight reporter construct, spCas9, and expression of sgRNAs, lentivirus was produced in HEK293T cells. HEK293T cells were transfected with lentiviral plasmids containing the gene of interest, pMD2.G plasmid, and PSPAX2 plasmid (Addgene #12259 and #12260, respectively) at a 2:1:1 ratio using 1 µl Lipofectamine-2000 (Thermo Fisher Scientific) per µg plasmid DNA. Culture medium was replaced after 18 h, and lentiviral supernatants were harvested 48 h later. After harvesting lentiviral supernatant were cleared from cells by a 10 min centrifugation at 500 × *g*, followed by filtration using a 0.45 µm syringe filter. Cells were transduced with lentiviral stocks overnight in the presence of 8 µg/ml polybrene (Sigma), after which the lentiviral medium was replaced with standard culture medium. Starting 24 h after lentiviral transduction, cells were cultured and expanded in the presence of their respective selection antibiotics. Cells lentivirally transduced with CROSS-FIRE and Cre-LoxP fluorescent Stoplight reporter constructs were sorted for eGFP^−^mCherry^+^ or eGFP^−^DsRed^+^ fluorescence, respectively, 2 weeks after expansion in the presence of selection antibiotics on a BD FACSAria III cell sorter. Stable MDA-MB-231 cell lines expressing Pol II-compatible sgRNA constructs were generated by linearized plasmid transfection using Lipofectamine-2000. pHAGE2-EF1a-sgRNA-UBC-Puro and pInducer20-sgRNA plasmids were linearized using SmaI or FseI restriction enzymes (New Englang Biolabs) respectively, followed by a DNA clean up using a PCR purification kit (Qiagen). Starting 48 h after transfection, cells were cultured and expanded in the presence of their respective selection antibiotics. The following concentrations of selection antibiotics were used: 2 µg/ml puromycin, 5 µg/ml blasticidine, or 500 µg/ml G418 for all cell types, with the exception of HEK293T cells being cultured with 1000 µg/ml G418.

### Co-cultures and transwell experiments

All co-culture experiments were performed in DMEM containing 10% FBS, l-Glutamine, 100 µg/ml streptomycin, and 100 µ/ml penicillin. Unless stated otherwise, direct co-culture experiments were performed for five days at a 1:5 ratio of reporter:donor cells. Transwell experiments were performed in 12-well plates for ten days, and after day five of transwell co-culture experiments, reporter cells were passaged to new wells, and new transwell inserts containing donor cells were added to the reporter cells. At the end of a co-culture experiment, cells were directly analyzed by fluorescence microscopy using an EVOS FL Cell Imaging System (Thermo Fisher Scientific), or analyzed by flow cytometry. For flow cytometry analysis cells were trypsinized for 5 min using TrypLE Express (Thermo Fisher Scientific), and transferred to 5 ml flow cytometry tubes using a similar volume of DMEM containing 10% FBS. Cells were centrifuged for 5 min at 300 × *g*, washed in 5 ml 1% FBS in PBS, and centrifuged once more for 5 min at 300 × *g*. Cells were then resuspended in 250 µl 1% FBS in PBS, and kept on ice until flow further analysis. Cells were analyzed on an ImageStream Mark II (Amnis), MacsQuant VYB (Miltenyi Biotec), or Fortessa (BD Biosciences) flow cytometer, and further analyzed using FlowJo v10 software.

### In silico confocal microscopy image analysis

Cells were seeded in CellStar 96-well cell culture black µClear bottom TC-treated microplates (Greiner-Bio). Prior to imaging, 1 µg/ml Hoechst 33342 (ThermoFisher Scientific) was added to the culture medium for 15 min at room temperature. Confocal pictures were then taken using a Yokogawa C7000 confocal microscope with a live cell stage incubator at 37 °C and 5% CO_2_. Sixteen images were taken at 20× magnification per well, at random locations using the following filter settings: Hoechst: emission = 405 nm, power 30; acquisition =BP445/45. Exposure time = 100. Input level = 2000. eGFP: emission = 488 nm, power 30; acquisition = BP525/50. Exposure time = 125. Input level = 10,000. mCherry: emission = 561 nm, power 30; acquisition = BP600/37. Exposure time = 125. Input level = 2000. Per picture, average fluorescence images were generated from z-stacks over a distance of 10 µm (5 µm below and 5 µm above nuclear focal point) with a 2 µm slice length. Images were then analyzed using the Columbus Image Data Storage and Analysis System (Perkin-Elmer), using the following settings: Find Nuclei: software analysis method “C”. Cytoplasm cell region selection: Region Type = nucleus region, Outer Border = −75%, Inner Border = −5%. mCherry+ selection: Population = all cells, Mean Cytoplasm Intensity BP600/37 > 110. eGFP+ selection: Population = mCherry+ cells, Mean Cytoplasm Intensity BP525/50 > 200.

### Luciferase activity assays

Cells were seeded in 96 well plates in 250 µl complete culture medium, 24 h prior to luciferase activity measurements. After 24 h, 150 µl conditioned medium was harvested to 1.5 ml Eppendorf tubes, and cleared of cellular debris by 5 min centrifugation at 300 × *g*, followed by 15 min centrifugation at 2000 × *g*. 75 microliter conditioned medium was then transferred to LumiNunc White 96-well plates (ThermoFisher Scientific), and Gaussia Luciferase activity was analyzed using the Dual-Glo Luciferase Assay System (Promega) according to the manufacturer’s protocol, and measured on a SpectraMax L Microplate Reader (Molecular Devices).

### EV isolation

For EV isolation, MDA-MB-231 cells expressing sgRNAs were cultured in CELLine Adhere 1000 Bioreactors (Integra Biosciences)^53–55^, in which MDA-MB-231 cells were maintained in a matrix concentrated cell compartment. This compartment is connected to an outer medium compartment through a 10 kDa pore-size semi-permeable membrane, which allows exchange of nutrients, but not extracellular vesicles, between both compartments. The outer medium compartment contained 500 ml complete culture medium with 0.5 µg/ml puromycin, and was changed on a weekly basis. The MDA-MB-231 cells were maintained in the cell compartment in 15 ml serum-free OptiMEM, which was replaced every 48 h. For EV isolation, the serum-free conditioned medium was isolated from the concentrated cell compartment and cell debris was removed by 5 min centrifugation at 300 × *g*, followed by 15 min centrifugation at 2000 × *g* ml. Samples were then filtered by 0.45 µm syringe filtration and further concentrated to a volume of 0.5–1.0 ml using a 100 kDa Amicon Ultra-15 Centrifugal filter (Merck). EVs were then isolated by size exclusion chromatography over a Tricorn 10/300 column with Sepharose 4 Fast Flow resin, using the AKTA Start chromatography system (all GE Healthcare Life Sciences). EV-containing fractions were sterilized by 0.45 µm syringe filtration, and concentrated using a 100 kDa Amicon Ultra-15 Centrifugal filter. Isolated EVs were directly used for functional assays, or stored at −20 °C until further analysis. For EV addition experiments on siRNA-treated reporter cells (see below), EVs were isolated from T175 flasks to facilitate high yield EV isolation every 24 h. For these isolation MDA-MB-231 sgRNA^+^ cells were cultured in T175 flasks in standard culture medium until a confluency of ~80% was reached. Cells were then washed once with OptiMEM, and cultured for 24 h in OptiMEM containing 100 µg/ml streptomycin, and 100 u/ml penicillin. Conditioned medium was then isolated, and cell debris was removed by 5 min centrifugation at 300 × *g*, followed by 15 min centrifugation at 2000 × *g*. Conditioned medium was then concentrated by tangential flow filtration using a Minimate 100 kDa Omega Membrane (Pall Corporation) to a volume of 15 ml, followed by 0.45 µm syringe filtration and further concentration to a volume of 0.5–1.0 ml using a 100 kDa Amicon Ultra-15 Centrifugal filter (Merck). EVs were then isolated by size exclusion chromatography, sterilized by syringe filtration and concentrated using 100 kDa centrifugal filters as described above. In experiments where soluble protein-containing fractions were isolated from conditioned medium alongside EVs by size exclusion chromatography, we made use of a Minimate 10 kDa Omega Membrane and 10 kDa Amicon Ultra-15 Centrifugal filters for sample concentration.

### Nanoparticle tracking analysis

EV size distribution was determined using a Nanosight S500 nanoparticle analyzer (Malvern Instruments) with a 405 nm laser. Samples were measured in PBS, with the camera setting at level 16. For post-acquisition analysis, all post-acquisition settings were set to “Auto”, with the exception of a fixed detection threshold of level 6. Using a scripted control function, five 60 s videos were recorded for each sample, and analyzed using NTA software v3.1.

### EV RNAse protection assay

50 µl EVs in PBS were mixed with 250 µl control (100 mM Tris, 5 mM EDTA, 200 mM NaCl) or lysis buffer (100 mM Tris, 5 mM EDTA, 200 mM NaCl, 0.2% SDS). Proteinase K (Thermo Fisher Scientific) was added to the appropriate samples at a final concentration 80 µg/ml. All samples were then incubated at 37 °C for 5 min, followed by Proteinase K heat inactivation of all samples at 90 °C for 5 min. After samples had cooled, RNase A (Thermo Fisher Scientific) was added to the relevant samples at a final concentration of 330 µg/ml, followed by a 20 min RNase digestion at 37 °C. RNase activity was halted by the addition of 900 µl Trizol LS (Life Technologies) and RNA was isolated according to the manufacturer’s protocol using GlycoBlue coprecipitant (Thermo Fisher Scientific). The RNA pellets were resuspended in 10 µl RNAse-free water, followed by cDNA synthesis using a SuperScript 3 kit (Thermo Fisher Scientific), RNasin Ribonuclease inhibitor (Promega) and 2 pmol of Targeting sgRNA reverse primer (See Supplementary Table 6), according to the manufacturer’s protocol. The resulting cDNA templates were then diluted 1:4 and PCR was performed by incubation at 95 °C for 2 min followed by 38 cycles of 95 °C for 30 s, 60 °C for 30 s, and 72 °C for 60 s on a C1000 Touch Thermal Cycler (Bio-Rad). Targeting sgRNA PCR primers are listed in Supplementary Table 6. PCR products were then run on a 1.5% agarose gel containing 1:10,000 GelRed DNA staining dye (Biotium) and imaged in the UGenius gel imaging system (Syngene). Uncropped gel scans have been included in the supplemental Source Data file.

### Western blot

Cells or EVs were lysed in RIPA buffer supplemented with Protease Inhibitor Cocktail (Sigma-Aldrich). Lysates were incubated on ice for 30 min and subsequently centrifuged for 15 min at 12,000 × *g* to remove non-soluble materials. Protein concentrations were determined by a MicroBCA Protein Assay, alongside a bovine serum albumin standard according to the manufacturer’s protocol (Thermo Fisher Scientific). Sample were mixed with sample loading buffer, where necessary containing 100 µM DTT, followed by a 10 min heat-inactivation at 95 °C. Samples were then loaded onto 4–12% gradient Bis–Tris polyacrylamide gels (Thermo Fisher Scientific) and subjected to electrophoresis. Proteins were then blotted onto Immobilon-FL polyvinylidine difluoride membranes (Millipore), which were subsequently blocked with in blocking buffer containing one part Odyssey Blocking Buffer (LI-COR Biosciences) and one part Tris-Buffered Saline (TBS). Membranes were subsequently probed using the following antibodies: Alix 1:1000 (Thermo Fisher Scientific, MA1-83977), Calnexin 1:1000 (GeneTex, GTX101676), CD9 1:1000 (Abcam, ab92726), CD63 1:1000 (AB8219), Flot-1 1:1000 (Cell Signaling Technology, 3253), TSG101 1:1000 (Abcam, ab30871), and H2B 1:1000 (Abcam, ab52599) in staining buffer consisting of one part Odyssey Blocking Buffer and one part TBS with 0.1% Tween-20 (TBST). Secondary antibodies consisted of either anti-rabbit IgG conjugated to AlexaFluor 680 (Thermo Fisher Scientific, A-21076) or anti-mouse IgG conjugated to IRDye 800CW and were applied at a 1:10,000 dilution in staining buffer. Proteins were visualized using an Odyssey Infrared Imager (LI-COR Biosciences) at 700 and 800 nm. Uncropped Western Blot scans have been included in the supplemental Source Data file.

### Transmission electron microscopy

EVs were adsorbed to carbon-coated formvar grids for 15 min at room temperature. Unbound EVs were removed by a PBS wash. Grids were then fixed in a 2% paraformaldehyde, 0.2% glutaraldehyde in PBS fixing buffer for 30 min at room temperature, followed by counterstaining with uranyl-oxalate. Grids were then embedded in a mixture of 1.8% methyl cellulose and 0.4% uranyl acetate at 4 °C. Grids were imaged on a Jeol JEM-1011 TEM microscope (Jeol).

### EV staining and uptake

EVs were fluorescently labeled with PKH67 (Sigma-Aldrich) as follows: Diluent C was added to EVs in a 1:1 v/v ratio, which were then labelled with PKH67 by the addition of one half additional volume of PKH67 diluted 1:100 in Diluent C. The mixture was incubated for 5 min at room temperature, after which unbound PKH67 dye was removed using the AKTA Start chromatography system (GE Healthcare Life Sciences) and a XK-16/20 column (GE Healthcare) with 30 ml of Sepharose CL-4B resin. After free dye removal, EV containing fractions were concentrated to 250 µl in 100 kDa Amicon Ultra-15 Centrifugal filter (Merck). 20 µl of labeled EVs were added of HEK293T Stoplight^+^ cells seeded on a gelatin coated glass 2-well chamber slide with removable wells (Thermo Fisher Scientific). After 6 h of incubation, the cells were fixed by the addition of paraformaldehyde and washed with PBS. Nuclei were stained for 10 min in 1 µg/ml DAPI. After fixation, slides were washed with PBS and mounted using Fluorsave (Calbiochem). Confocal fluorescence imaging was performed using a LSM700 laser scanning confocal microscope (Zeiss). Images were processed using LSM Image Browser.

### EV addition experiments

For comparison of EVs containing targeting sgRNAs or non-targeting sgRNAs, and for EV dose response addition experiments, HEK293T Stoplight^+^spCas9^+^ reporter cells were cultured in 24-well plates in 1 ml culture medium, and EVs isolated from MDA-MB-231 cells expressing sgRNAs cultured in CELLine Adhere 1000 Bioreactors (Integra Biosciences) were added. For EV addition experiments on siRNA-treated reporter cells, cells were plated in 96-well plate wells in a volume of 200 µl, and EVs isolated from MDA-MB-231 cells expressing sgRNAs cultured in T175 flasks were added every 24 h for six consecutive days. After EV addition, EVs were incubated with the cells for 24 h, followed by a culture medium wash. Cell confluences between 40 and 100% were maintained throughout the addition experiment. On average, 1.5e12 EVs per 1e5 cells were added in EV addition experiments. For RNase A treatments, EVs were incubated in PBS for 30 min at 37 °C with 10 µg/ml RNAse A prior to addition to cells. After the last EV addition, reporter cells were incubated for another 48 h to allow reporter cells activated in the last addition to reach sufficient eGFP levels. Reporter cells were analyzed by fluorescence microscopy and flow cytometry as described above.

### siRNA knockdown

Cells were seeded in DMEM containing 10% FBS, l-Glutamine, and no antibiotics, 24 h prior to siRNA transfection, in 24-well plates. Cells were transfected using Lipofectamine RNAiMax (Life Technologies) according to the manufacturer’s protocol. 5 or 1.25 pmol siRNAs previously verified^12^, or Dharmacon ON-TARGETplus siRNA smartpools were transfected in 24-well or 96-well plate wells, respectively. Annealed siRNA sequences are listed in Supplementary Table 4, Dharmacon ON-TARGETplus siRNA smartpool and non-coding siRNA control product information is listed in Supplementary Table 5. Prior to any functional experiments, target gene knockdown (KD) of all used siRNAs was confirmed by qPCR in the appropriate cell types (Supplementary Fig. 10). After 18 h, cells were washed once, and subsequently cultured in DMEM containing 10% FCS, l-Glutamine, 100 µg/ml streptomycin, and 100 u/ml penicillin. In case of co-culture experiments, additional cells were added directly after the culture medium was changed. For qPCR analysis, cells were cultured for an additional 48 h before RNA isolation.

### Cell RNA isolation and qPCR analysis

Total RNA was isolated from cells using TRIzol Reagent (Thermo Fisher Scientific), according to the manufacturer’s protocol. Isolated RNA was measured using a DS-11 Spectrophotometer (DeNovix). One µg RNA per sample was used for cDNA synthesis using the iScript cDNA Synthesis Kit (Bio-Rad). qPCR was performed using iQ SYBR Green Supermix (Bio-Rad) in a CFX96 Real‐Time PCR Detection System (Bio‐Rad). Primer sequences were taken from the PrimerBank PCR primer public resource^56^ and synthesized by Integrated DNA Technologies (Supplementary Table 6). Cycle threshold (Ct) values were normalized per experiment and per gene. ΔΔCt was calculated using housekeeping gene GAPDH. Statistical analysis was performed using a Student’s *t*-test.

### Quantification of sgRNA abundance in EVs

EVs were isolated from sgRNA^+^ MDA-MB-231 donor cells as described above. EV count was determined using NTA as described above. 250 µl of the EV samples were lysed in 750 µl trizol LS (Thermo Fisher Scientific) and RNA was extracted using glycoblue co-precipitant (Thermo Fisher Scientific) as per manufacturer’s instructions. Reverse transcription was then performed using a superscript IV reverse transcriptase kit (Thermo Fisher Scientific) and a targeting RNA specific reverse primer. A standard curve was prepared which contained synthetic targeting sgRNA at known copy numbers in 250 µl PBS. RNA was extracted from these standard curve samples and reverse transcription was performed using an identical method alongside EV samples in order to normalize for RNA extraction and reverse transcription efficiency. A PBS-only blank was also taken along to rule out contamination. qPCR was then performed on the standard curve, targeting EV and blank cDNA samples. The targeting sgRNA copy number in EV samples was then interpolated from the standard curve Ct values using Graphpad Prism 8.0.1 software.

### Statistics

All statistical analyses were performed using Graphpad Prism 8.01. Values are expressed as the mean ± standard deviation (SD), unless indicated otherwise. Two-sided statistical tests were performed in all statistical analyses. Differences were considered statistically significant at *p* < 0.05.

### Reporting summary

Further information on research design is available in the Nature Research Reporting Summary linked to this article.

## Supplementary information

Supplementary Information

Reporting Summary

## Data Availability

The datasets generated during and/or analyzed during the current study are available from the corresponding author on reasonable request. The source data underlying Figs. 1c, f–g, 2c–e, 3b–c, e, h–i, 4b–c, e–g, 5d, e, and Supplementary Figs. 1B–C, 3C–F, 4B, D, F, H, J, 5B, D, F, 6B, D–F, 7B–E, 8A–C, 9B, D, F, and 10A–C are provided as a Source Data file.

## Source Data

Source Data
