# Publisher Correction: A CRISPR-Cas9-based reporter system for single-cell detection of extracellular vesicle-mediated functional transfer of RNA

**DOI:** 10.1038/s41467-020-15347-0

**Published:** 2020-03-31

**Authors:** Olivier G. de Jong, Daniel E. Murphy, Imre Mäger, Eduard Willms, Antonio Garcia-Guerra, Jerney J. Gitz-Francois, Juliet Lefferts, Dhanu Gupta, Sander C. Steenbeek, Jacco van Rheenen, Samir El Andaloussi, Raymond M. Schiffelers, Matthew J. A. Wood, Pieter Vader

**Affiliations:** 1 Laboratory of Clinical Chemistry and Hematology, University Medical Center Utrecht, Utrecht, The Netherlands; 2 Department of Paediatrics, University of Oxford, Oxford, United Kingdom; 3 Department of Physiology, Anatomy and Genetics, University of Oxford, Oxford, United Kingdom; 4 Department of Physics, University of Oxford, Oxford, United Kingdom; 5 Pediatric Pulmonology and Regenerative Medicine Center, University Medical Center Utrecht, Utrecht, The Netherlands; 6 Department of Laboratory Medicine, Clinical Research Center, Karolinska Institutet, Huddinge, Sweden; 7 Department of Molecular Pathology, Oncode Institute, Netherlands Cancer Institute, Amsterdam, The Netherlands; 8 Department of Experimental Cardiology, University Medical Center Utrecht, Utrecht, The Netherlands

**Keywords:** Nanoparticles, Assay systems, Membrane trafficking

Correction to: *Nature Communications* 10.1038/s41467-020-14977-8, published online 28 February 2020.

The original version of this Article contained errors in Fig. 4; in panel b the Rab27A column was incorrectly labelled as Rab24A, and in panel f the VAV2 column was incorrectly labelled as VAn2. These have been corrected in both the PDF and HTML versions of the Article.

