# Supplementary material for: A CRISPR-Cas9-based reporter system for single-cell detection of extracellular vesicle-mediated functional transfer of RNA
Source: Nat Commun. 2020 Feb 28;11:1113. doi: 10.1038/s41467-020-14977-8 (PMC7048928; doi:10.1038/s41467-020-14977-8)
Supplement: Supplementary file 1 — Supplementary Information [file 41467_2020_14977_MOESM1_ESM.pdf]

A CRISPR-Cas9-based reporter system for single-cell detection of extracellular vesicle-mediated functional transfer of RNA

Olivier G. de Jong, Daniel E. Murphy, Imre Mäger, Eduard Willms, Antonio Garcia-Guerra, Jerney J. Gitz-Francois, Juliet Lefferts, Dhanu Gupta, Sander C. Steenbeek, Jacco van Rheenen, Samir El Andaloussi, Raymond M. Schiffelers, Matthew J.A. Wood, Pieter Vader

Supplementary Figures, Tables, and References

Supplementary Figure 1

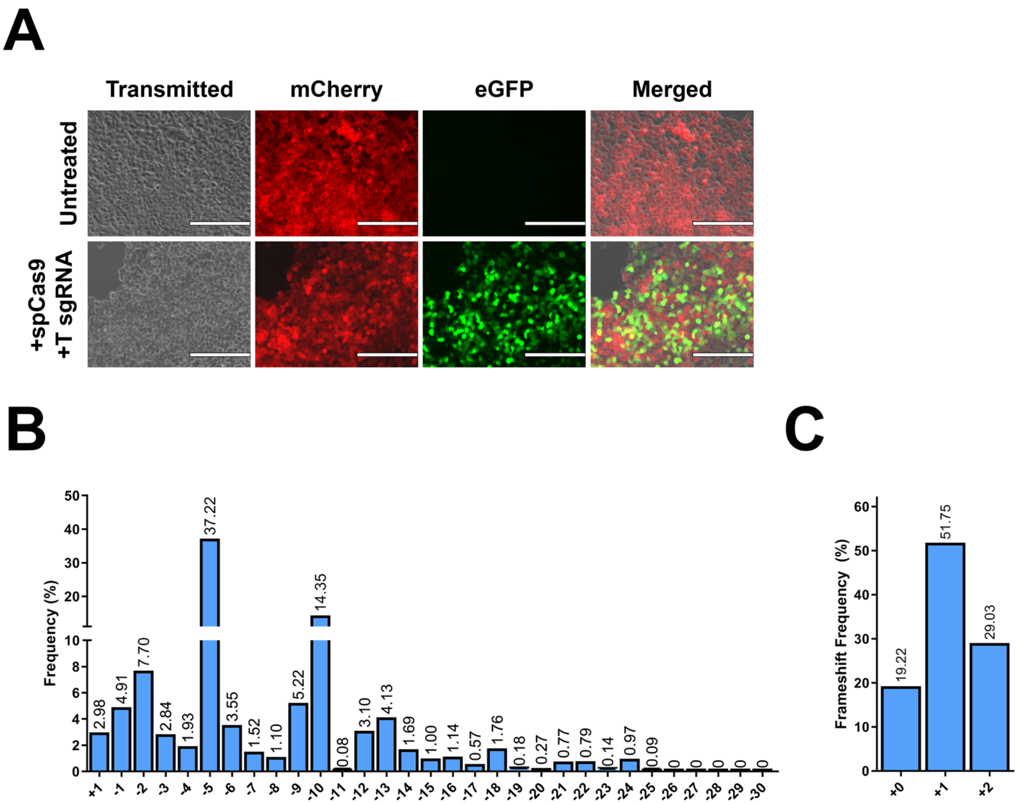

Supplementary Figure 1 | Characterization of the Cas9 Non-Homologous End Joining (NHEJ) Fluorescent Stoplight reporter. a, Fluorescent microscopy images of stable HEK293T Stoplight+ cells left untreated (top row), or 72 hrs after transfection with plasmids encoding for spCas9 and a sgRNA targeting the stoplight construct. Scale bar represents 200 µm. Representative images as observed in 3 biologically independent samples. **b, c**, inDelphi *in silico* CRISPR editing prediction[1] of spCas9 in HEK293 cells using a 5-‘ ACTCCGATCGGAGGACAGTACTCCGCTCG-Cut Site- AGTCGGCTAGCGGGCCCGGGGTTGC-3’ prediction sequence showing predictions for indel frequency (**b**) and frameshift frequency (**c**). Both +1nt and +2nt frameshifts result in permanent activation of eGFP expression.

Supplementary Figure 2

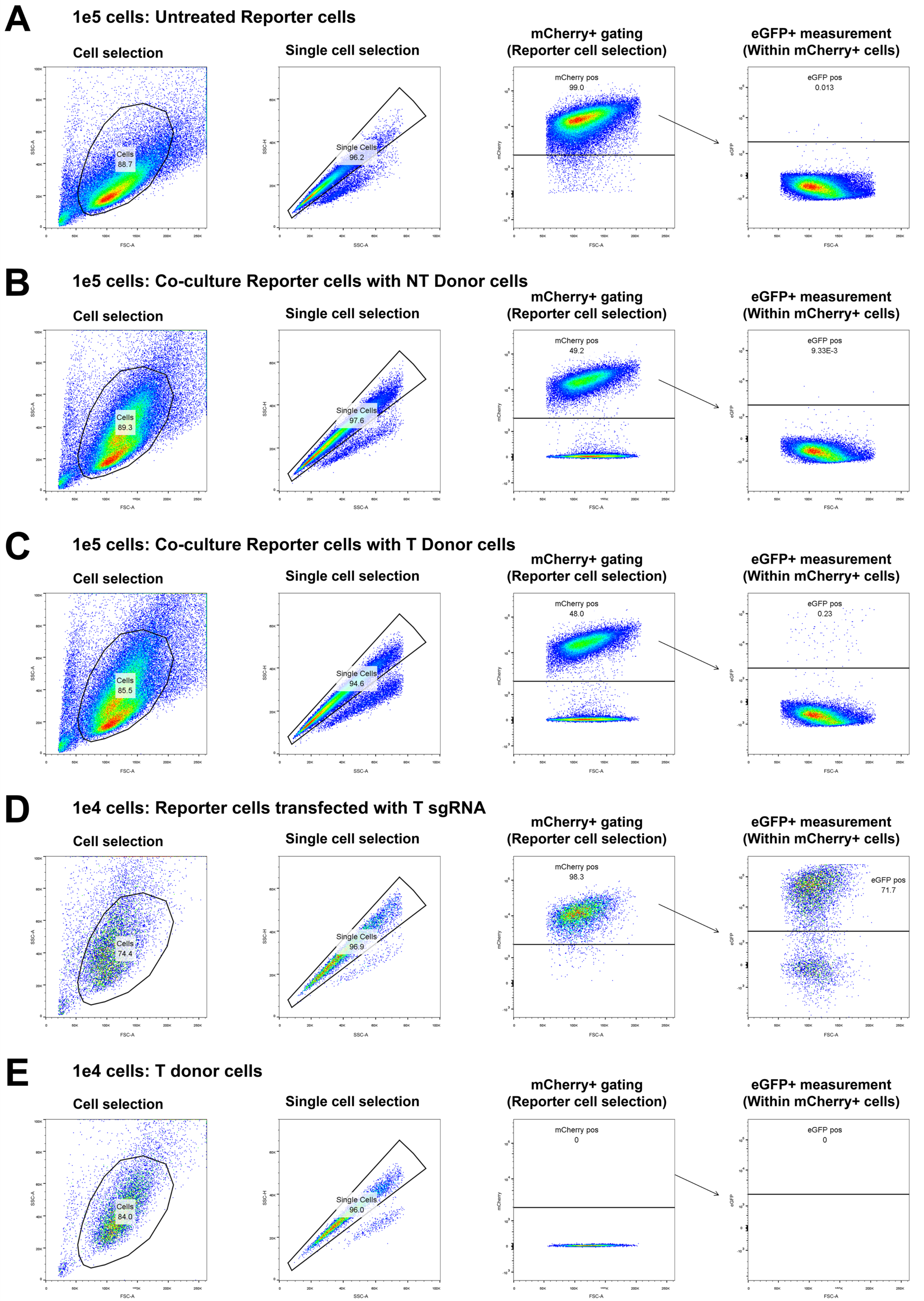

Supplementary Figure 2 | Flow cytometry gating strategy employed to analyze expression and activation of the Fluorescent Stoplight reporter construct. First, cells are gated using forward scatter area (FSC-A) and sideward scatter area (SSC-A). Then, single cells are selected based on SSC-A and sideward scatter height (SSC-H) signals. Thirdly, Stoplight$^+$ reporter cells are measured and gated using FSC-A and mCherry signals. Lastly, eGFP$^+$ cells are measured within mCherry+ cells FSC-A and eGFP signals. Representative flow cytometry plots are shown for untreated HEK293T Stoplight$^+$spCas9$^+$ reporter cells (**a**), A five day co-culture of HEK293T Stoplight+spCas9+ reporter cells with sgRNA$^+$ MDA-MB-231 donor cells expressing a non-targeting (NT) sgRNA (**b**), or a targeting (T) sgRNA (**c**), HEK293T Stoplight$^+$spCas9$^+$ reporter cells 3 days after transfection with a plasmid encoding a targeting sgRNA (**d**), and untreated sgRNA$^+$ MDA-MB-231 donor cells (**e**), all from within the same experiment. Representative data as observed in 17 independent experiments.

Supplementary Figure 3

A

Detect nuclei based on Hoechst signal
↓
Determine area surrounding nuclei to select cytoplasma
↓
Quantify mCherry signal in cytoplasma
↓
Quantify number of mCherry+ cells
↓
Quantify eGFP signal in cytoplasma
↓
Quantify number of eGFP+ cells gated within mCherry+ cells

B

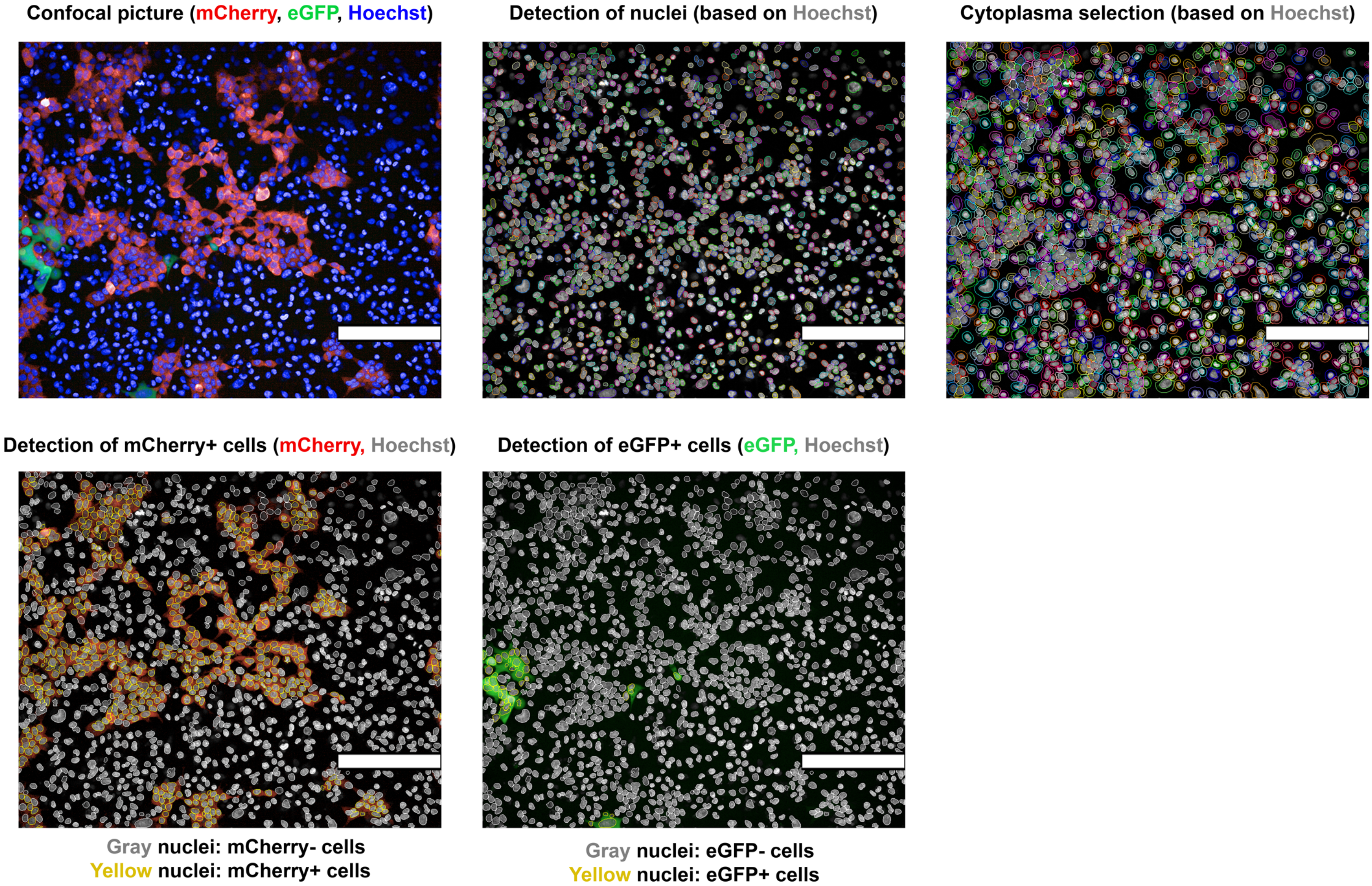

C **D** **E** **F**

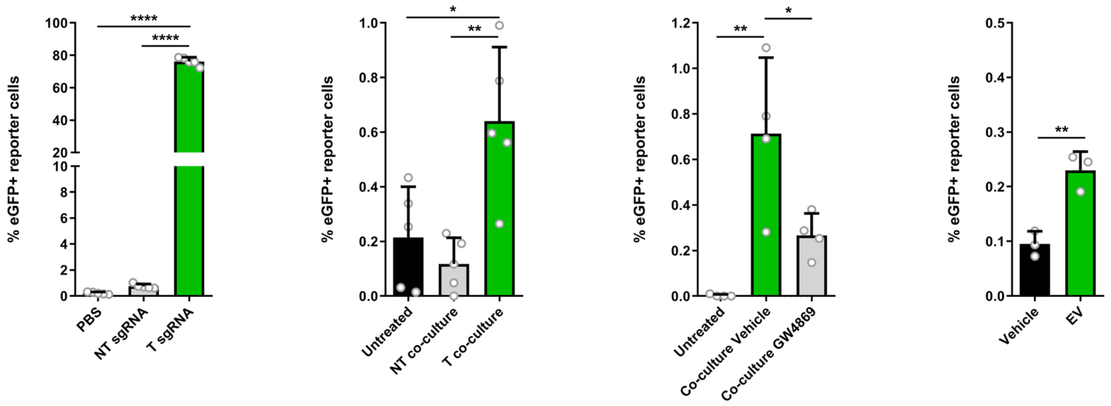

Supplementary Figure 3 | *In silico* confocal microscopy image analysis of the CROSS-FIRE system. Confocal images were analyzed using the Columbus Image Data Storage and Analysis System. A workflow of the analysis is shown in (**a**). **b**, Representative images of a five day co-culture of HEK293T Stoplight+spCas9+ reporter cells with sgRNA$^+$ MDA-MB-231 donor cells are shown to demonstrate the *in silico* analysis. From left to right, top to bottom, the following analyses are presented: 1, a merged confocal microscopy picture showing mCherry (red), eGFP (green) and Hoechst (blue); 2, detection of nuclei based on nuclear staining (Hoechst, gray); 3, selection of the areas directly surrounding the detected nuclei (Hoechst, gray) serves as a cytoplasm selection tool; 4, detection of nuclei (Hoechst, gray) positive for mCherry (red) in their respective cytoplasm region. Nuclei of mCherry-positive cytoplasm regions are marked in yellow; 5, detection of nuclei (Hoechst, gray) positive for eGFP (green) in their respective cytoplasm region. Nuclei of eGFP-positive cytoplasm regions are marked in yellow. Scale bar = 200 µm. **c** Confocal microscopy image analysis of HEK293T Stoplight$^+$spCas9$^+$ cells after transfection of a plasmid encoding a sgRNA targeting the Stoplight construct (+T sgRNA) or a non-targeting sgRNA (NT sgRNA) 3 days after transfection. Means + SD, n = 5 independent experiments, Tukey's multiple comparison test. **d**, Confocal microscopy image analysis of a five day co-culture of HEK293T Stoplight$^+$spCas9$^+$ reporter cells with MDA-MB-231 sgRNA$^+$ donor cells expressing a targeting sgRNA (T), and a non-targeting sgRNA (NT). Means + SD, n = 5 independent experiments, Tukey's multiple comparison test. **e**, Confocal microscopy image analysis of a five day direct co-culture experiment with MDA-MB-231 sgRNA+ donor cells with or without the presence of EV release inhibitor GW4869 at a concentration of 1 µM. Means + SD, n = 4 independent experiments, Tukey's multiple comparison test. **f**, Confocal microscopy image analysis of EV-mediated activation of the CROSS-FIRE platform using EVs isolated from sgRNA$^+$ MDA-MB-231 donor cells, as compared to vehicle-treated reporter cells (PBS). EVs were added every 24 hrs for 6 additions with an average concentration of 2.2e11$\pm$6.1e10 EVs per addition. Means + SD, n = 3 biological replicates, treated with EVs from parallel isolations, Tukey's multiple comparison test. * = p < 0.05, ** = p < 0.01, **** = p <0.0001.

Supplementary Figure 4

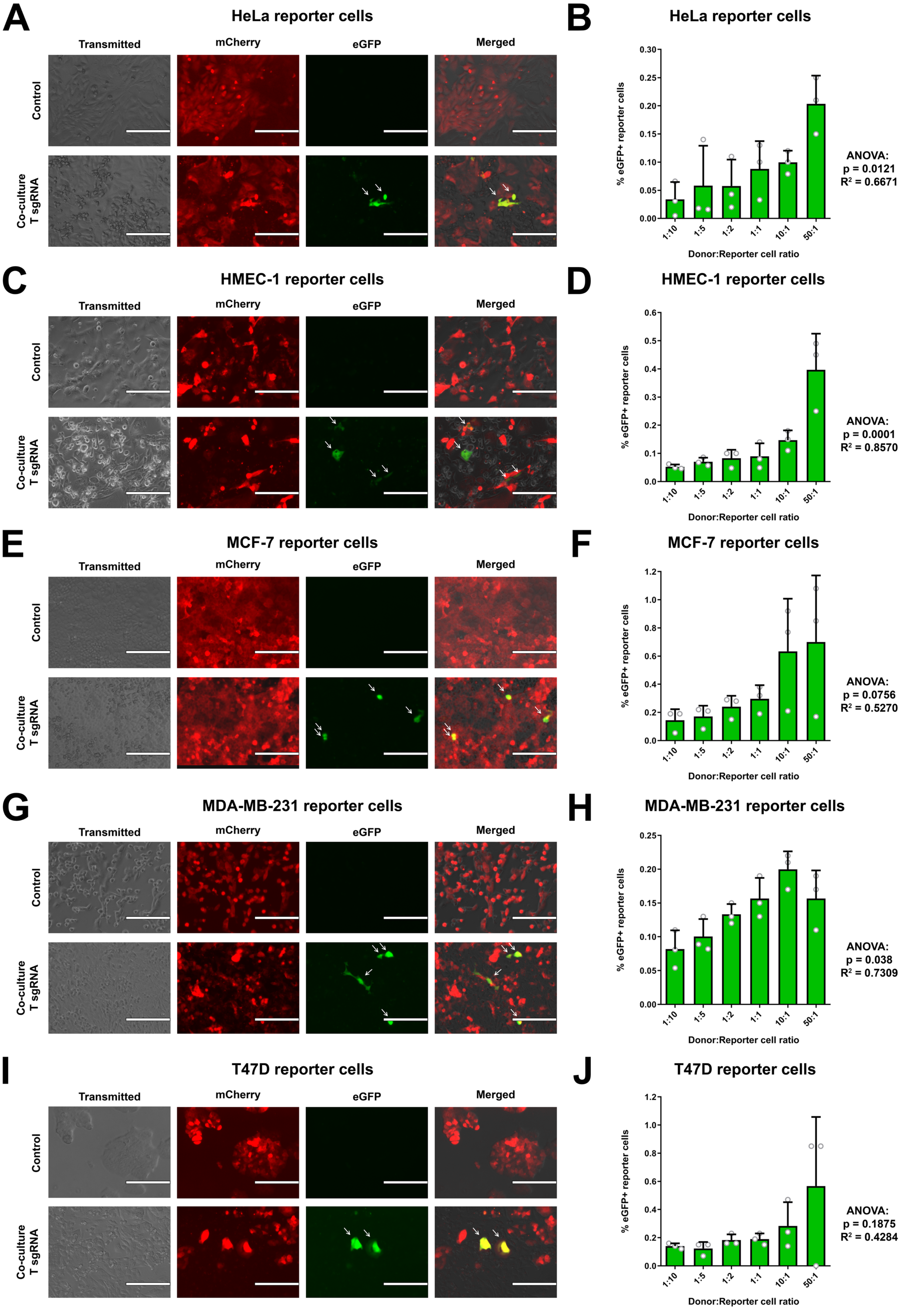

Supplementary Figure 4 | Functional intercellular sgRNA transfer to multiple additional reporter cell lines. sgRNA$^+$ MDA-MB-231 donor cells were cultured in various donor : reporter cell ratios with multiple additional Stoplight$^+$spCas9$^+$ reporter cell lines for five days: HeLa (**a**,**b**), HMEC-1 (**c**,**d**), MCF-7 (**e**,**f**), MDA-MB-231 (**g**,**h**) and T47D (**i**,**j**). Co-cultures were analyzed by fluorescence microscopy (**a**,**c**,**e**,**g**,**i**) and flow cytometry (**b**,**d**,**f**,**h**,**j**). Scale bar represents 200 µm. Means + SD, n =3 independent experiments, ANOVA.

Supplementary Figure 5

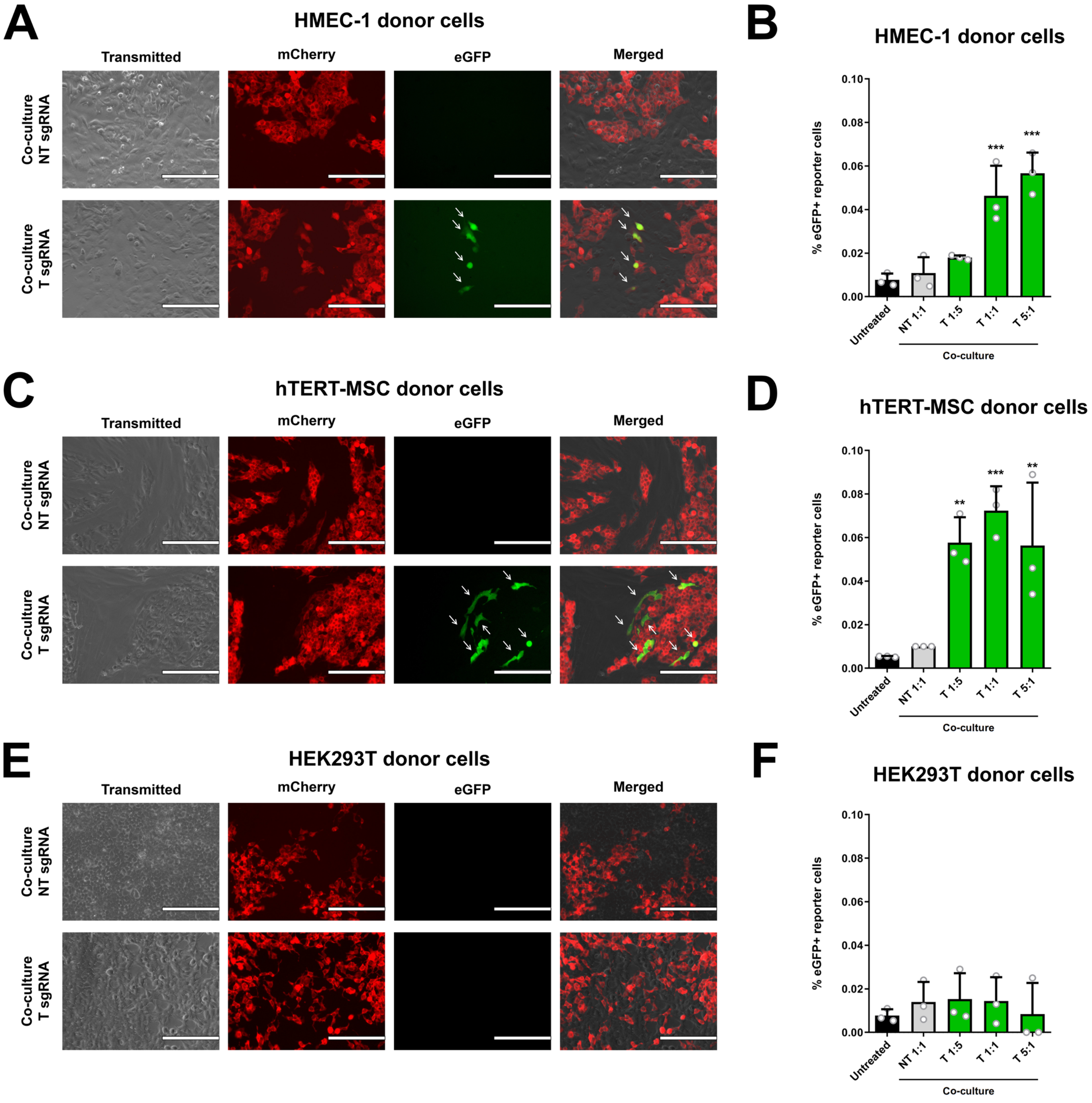

Supplementary Figure 5 | Functional intercellular sgRNA transfer by multiple additional donor cell lines. Stoplight$^+$spCas9$^+$ HEK293T reporter cells were cultured with various sgRNA$^+$ donor cell lines in a multiple ratios expressing targeting or non-targeting sgRNAs for five days: HMEC-1 (**a**,**b**), hTERT-MSC (**c**,**d**), and HEK293T (**e**,**f**).Co-cultures were analyzed by fluorescence microscopy (**a**,**c**,**e**) and flow cytometry (**b**,**d**,**f**). Scale bar represents 200 µm. Means + SD, n = 3 independent biological samples, Dunnett's multiple comparison test. ** = p < 0.01, *** = p <0.001.

Supplementary Figure 6

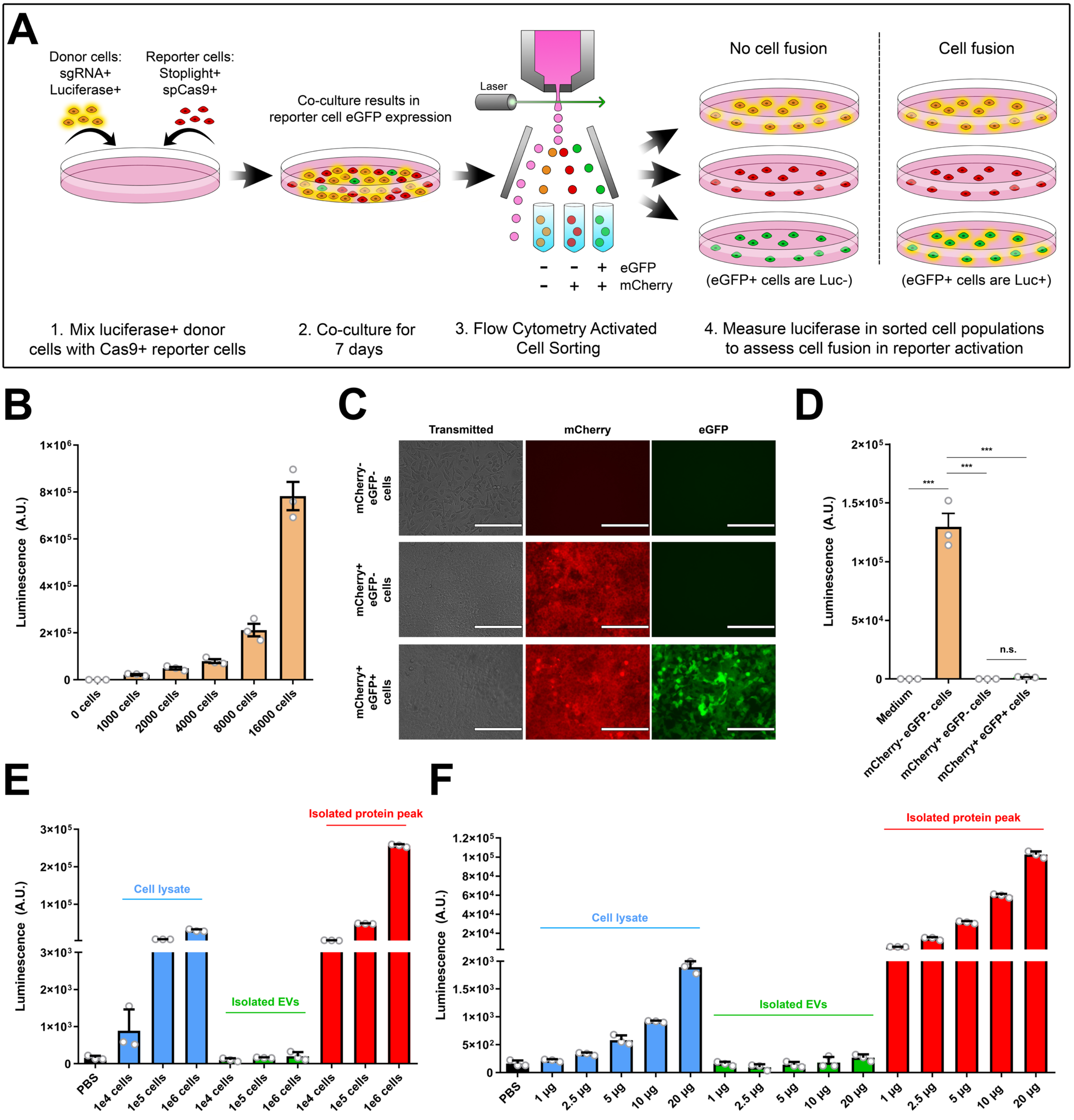

Supplementary Figure 6 | CROSS-FIRE reporter activation is not mediated by cellular fusion. **a**, Schematic of the experimental set up to study the potential role of cellular fusion in reporter activation. Gaussia Luciferase$^+$sgRNA$^+$ MDA-MB-231 donor cells and Stoplight$^+$spCas9$^+$ reporter cells are cultured in standard co-culture conditions for 7 days, resulting in reporter cell activation (eGFP expression). Cells are then harvested and subjected to fluorescence activated cell sorting in which 3 cell populations will be collected: donor cells (eGFP$^-$mCherrry$^-$), non-activated reporter cells (eGFP$^-$mCherry$^+$), and activated reporter cells (eGFP$^+$mCherry$^+$). These 3 populations are then analyzed for luciferase activity. If reporter activation is not the result of cellular fusion, only the eGFP$^-$mCherry$^-$ population will display luciferase activity (section 4, left), whereas cellular fusion would also result in luciferase activity in the eGFP$^+$mCherry$^+$ population (section 4, right). **b**, Confirmation of luciferase activity in the supernatant of Gaussia Luciferase$^+$sgRNA$^+$ stable MDA-MB-231 donor cells. Means + SD, n = 3 technical replicates, data representative of 2 independent experiments. **c**, Fluorescence microscopy pictures of plated eGFP$^-$mCherrry$^-$, eGFP$^-$mCherry$^+$, and eGFP$^+$mCherry$^+$ cell populations isolated by fluorescence activated cell sorting after a 7-day co-culture. Scale bar represents 200 µm. **d**, A luciferase activity assay on conditioned medium of 10,000 plated eGFP$^-$mCherrry$^-$, eGFP$^-$mCherry$^+$, and eGFP$^+$mCherry$^+$ cells after a seven day co-culture only shows luciferase activity in eGFP$^-$mCherry$^-$ cells. Means + SD, n = 3 biological replicates, Tukey's multiple comparison test. **e, f**, A luciferase activity assay on Gaussia Luciferase$^+$sgRNA$^+$ MDA-MB-231 donor cell lysates, alongside EVs and proteins isolated simultaneously from conditioned medium by size exclusion chromatography using 10 kDa tangential flow filtration and kDa Amicon Ultra-15 Centrifugal filters, as normalized for producing cell count (**e**) or BCA protein measurement (**f**). Means + SD, n = 3 technical replicates. *** = p <0.001.

Supplementary Figure 7

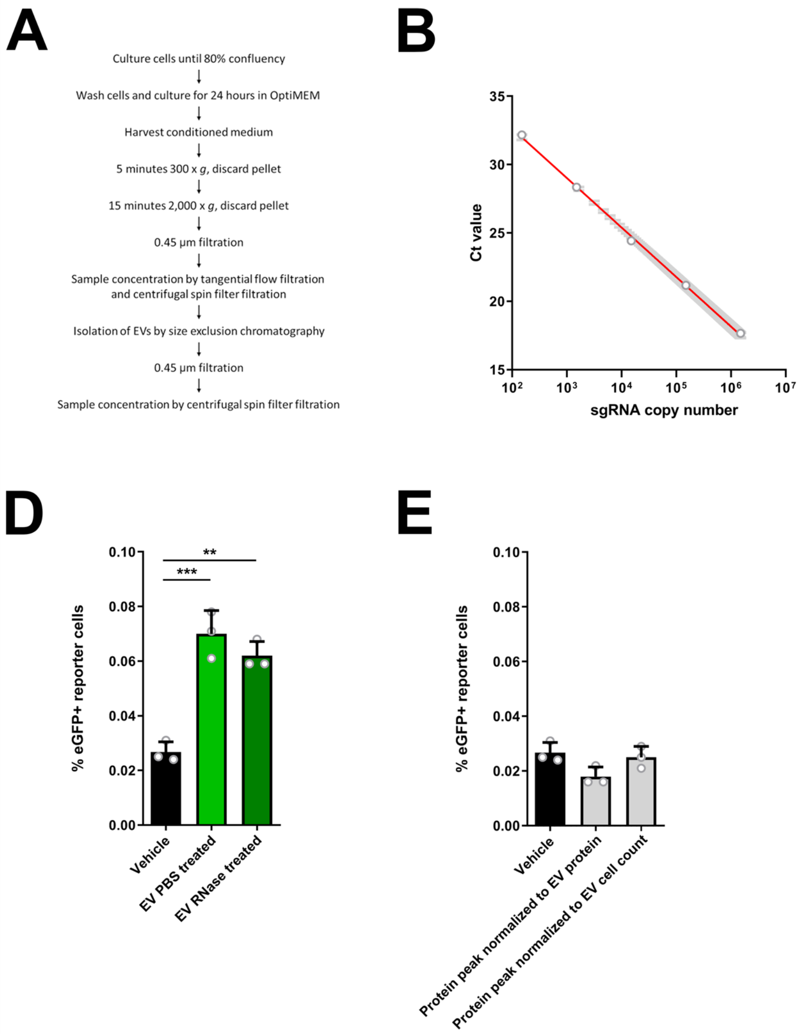

Supplementary Figure 7 | Characterization of EV-associated sgRNA. a, Overview of the applied workflow for EV isolation. **b**, qPCR of a concentration curve of synthetic CROSS-FIRE targeting sgRNAs after having undergone TRIzol RNA isolation and cDNA synthesis equal to, and alongside, RNA isolation and cDNA synthesis from sgRNA$^+$ MDA-MB-231-derived EVs. **c**, Quantification of the amount of sgRNA per sgRNA$^+$ MDA-MB-231-derived EV, based on qPCR alongside the sgRNA concentration curve (**b**) and NTA Nanosight Nanoparticle Tracking Analysis (NTA). Means + SD, n = 3 independent biological samples. **d,e**, Flow cytometry analysis of activation of the CROSS-FIRE platform using EVs (**d**) or the soluble protein-containing fractions (**e**) isolated from sgRNA$^+$ MDA-MB-231 donor cells by size exclusion chromatography using 10 kDa tangential flow filtration and 10 kDa Amicon Ultra-15 Centrifugal filters, as compared to vehicle-treated reporter cells (PBS). EVs were incubated for 30 minutes at 37° C in PBS with or without 10 µg/ml RNase A, and were added every 24 hrs for 6 additions with an average concentration of 2.2e11+6.1e10 EVs per addition. Added protein was either normalized for EV protein content based on BCA protein measurement, or for producing cell count. Means + SD, n = 3 biological replicates, Tukey's multiple comparison test. ** = p < 0.01, *** = p <0.001.

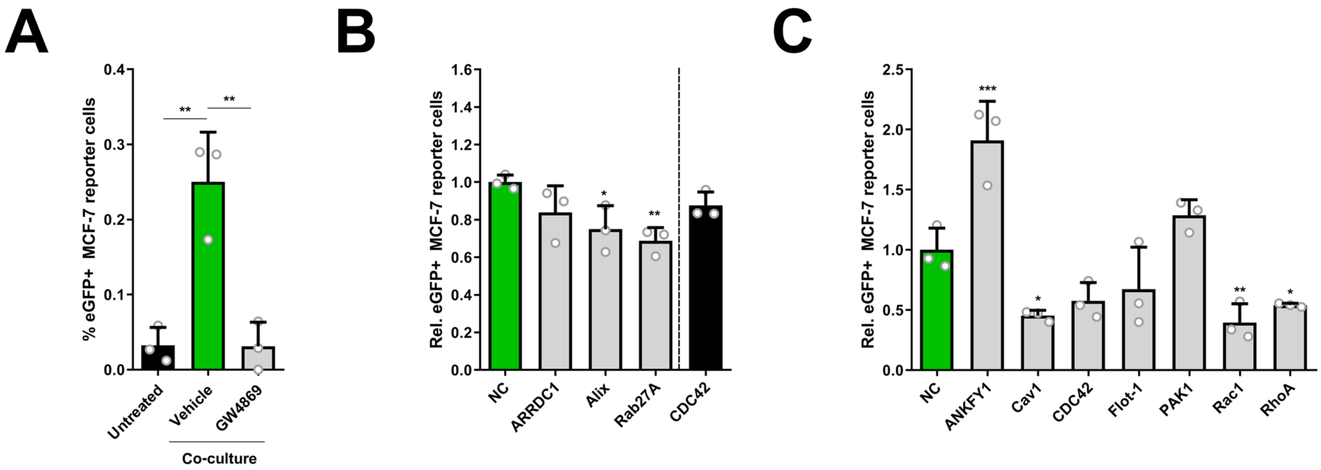
A
B
C
% eGFP+ MCF-7 reporter cells
Rel. eGFP+ MCF-7 reporter cells
Untreated
Vehicle
GW4869
Co-culture
**
**
NC
ARRDC1
Alix
Rab27A
CDC42
*
**
ANKFY1
Cav1
CDC42
Flot-1
PAK1
Rac1
RhoA
***
*
**
*

Supplementary Figure 8 | Confirmation of EV-mediated sgRNA transfer in Stoplight$^+$spCas9$^+$ MCF-7 reporter cells. **a**, Flow cytometry analysis of a five day direct co-culture experiment with EV release inhibitor GW4869 at a concentration of 1µM. Means + SD, n = 3 independent experiments, Tukey's multiple comparison test. **b**, Flow cytometry analysis of HEK293T Stoplight$^+$spCas9$^+$ reporter cells after a five day co-culture with sgRNA$^+$ MDA-MB-231 donor cells subjected to siRNA-mediated KD of ARRDC1, Alix, Rab27A, and CDC42, alongside a non-coding control siRNA (NC). Means + SD, n = 3 independent experiments, Dunnett's multiple comparison test. **c**, Flow cytometry analysis of MCF-7 reporter cells after a five day co-culture with sgRNA$^+$ MDA-MB-231 donor cells, in which the reporter cells were subjected to siRNA-mediated KD of ANKFY1, Cav1, CDC42, Flot-1, PAK1, Rac1, and RhoA alongside a non-coding control siRNA (NC). Means + SD, n = 3 independent experiments, Dunnett's multiple comparison test. * = $p < 0.05$, ** = $p < 0.01$, *** = $p < 0.001$.

Supplementary Figure 9

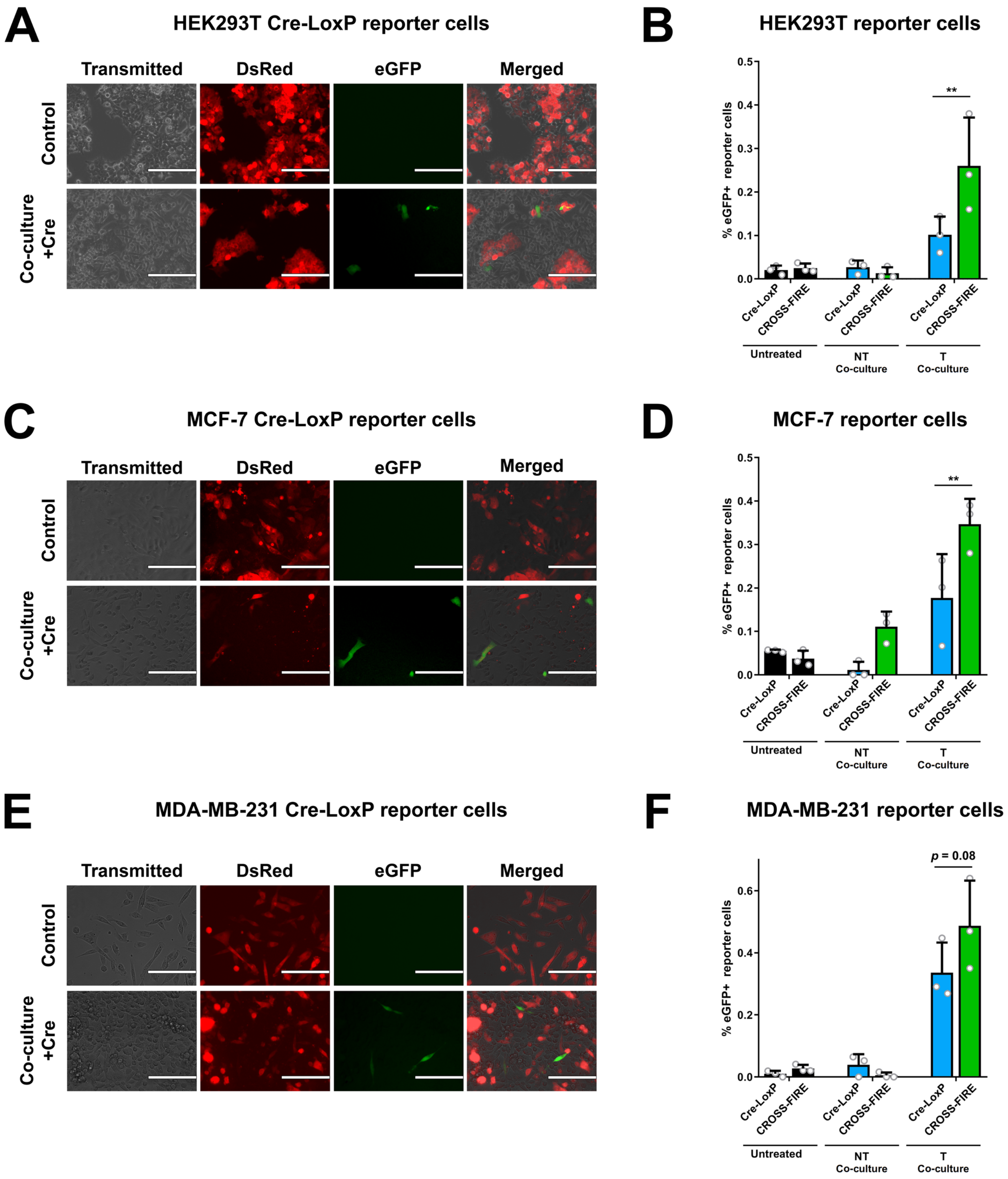

Supplementary Figure 9 | Analysis of the Cre-LoxP reporter system for EV cargo transfer. a,c,e, Fluorescence microscopy pictures of HEK293T (**a**), MCF-7 (**c**), or MDA-MB-231 (**e**) Cre-LoxP reporter cells (CMV-DsRed-LoxP-eGFP-LoxP) cultured for five days without (Control) or co-cultured with Cre^+ MDA-MB-231 donor cells. Scale bar = 200 µm. **b,d,f**, Flow cytometry analysis of HEK293T (**b**), MCF-7 (**d**), or MDA-MB-231 (**f**) $Stoplight^+spCas9^+$ reporter cells (CROSS-FIRE) or Cre-LoxP reporter cells after a five day direct co-culture experiment with their respective MDA-MB-231 non-targeting (NT) or targeting (T) donor cells. CROSS-FIRE and Cre-LoxP donor cells were used as NT donor cells for the opposite reporter system, respectively. Means + SD, n =3 biologically independent samples, Sidak's multiple comparisons test. ** = $p < 0.01$.

Supplementary Figure 10

A

B

C

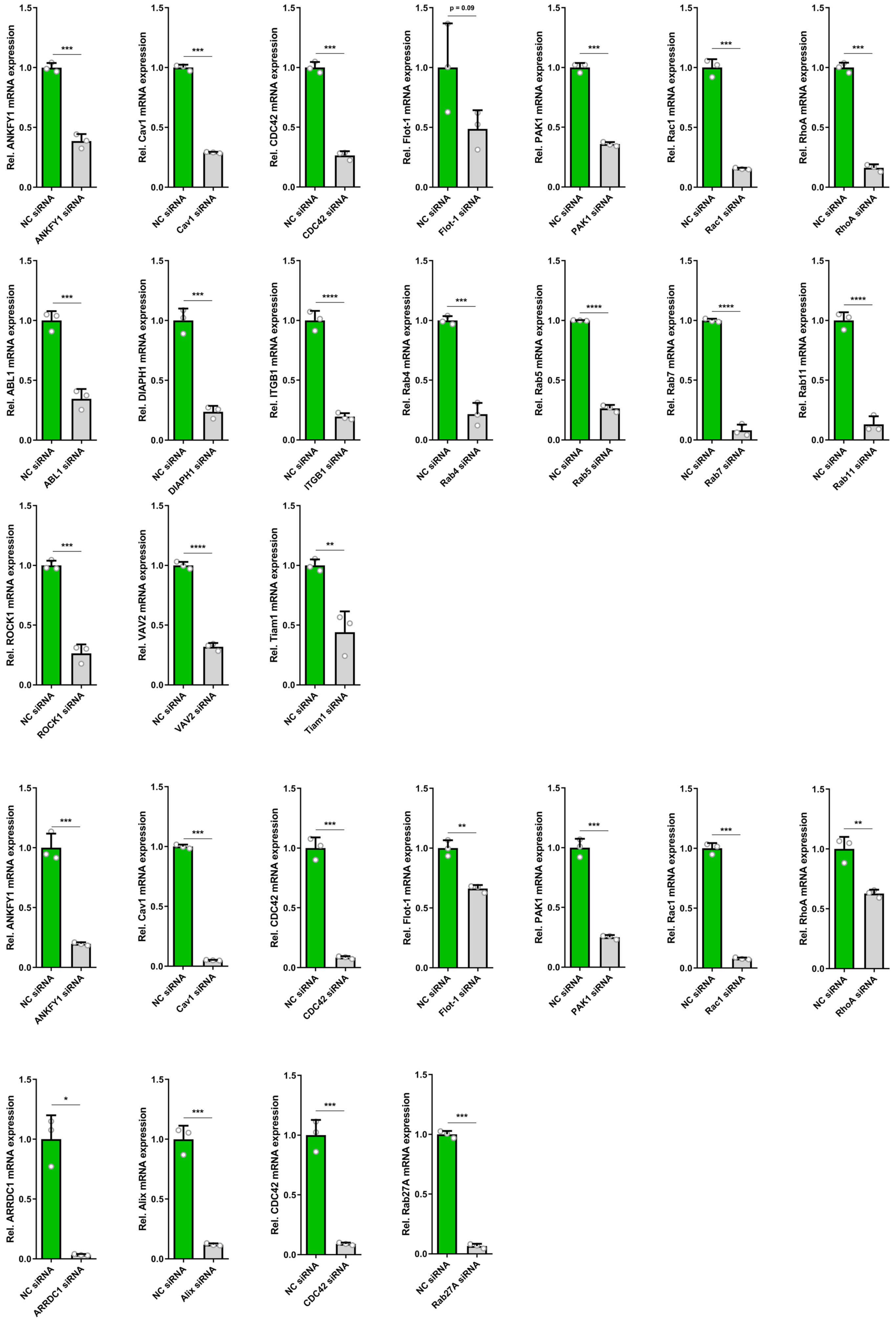

Supplementary Figure 10 | Confirmation of siRNA-mediated gene knockdown by qPCR. **a**, qPCR analysis of Stoplight$^+$Cas9$^+$ HEK293T cells transfected with siRNAs targeting genes involved in the regulation of EV uptake, 72 hours after siRNA transfection. Gene expression levels were normalized to GAPDH housekeeping levels. Means + SD, n = 3 biologically independent samples, Student's t-test. **b**, qPCR analysis of Stoplight$^+$Cas9$^+$ MCF-7 cells transfected with siRNAs genes involved in the regulation of EV uptake, 72 hours after siRNA transfection. Gene expression levels were normalized to GAPDH housekeeping levels. Means + SD, n = 3 biologically independent samples, Student's t-test. **c,** qPCR analysis of sgRNA$^+$ MDA-MB-231 cells transfected with siRNAs targeting genes involved in EV release and tunneling nanotube formation, 72 hours after siRNA transfection. Gene expression levels were normalized to GAPDH housekeeping levels. Means + SD, n = 3 biologically independent samples, Student's t-test. * = $p < 0.05$, ** = $p < 0.01$, *** = $p <0.001$, **** = $p <0.0001$.

Supplementary Table 1: Stoplight reporter construct:

Stoplight reporter construct	atggtgagcaagggcgaggaggataacatggccatcatcaaggagttcatgcgcttcaaggtgcacatggagggctccgtgaacggcc<br>acgagttcgagatcgagggcgagggcgagggccgcccctacgagggcacccagaccgccaagctgaaggtgaccaagggtggcccc<br>ctgcccttcgcctgggacatcctgtcccctcagttcatgtacggctccaaggcctacgtgaagcaccccgccgacatccccgactacttga<br>agctgtccttccccgagggcttcaagtgggagcgcgtgatgaacttcgaggacggcggcgtggtgaccgtgacccaggactcctccctg<br>caggacggcgagttcatctacaaggtgaagctgcgcggcaccaacttcccctccgacggccccgtaatgcagaagaagacgatgggct<br>gggaggcctcctccgagcggatgtaccccgaggacggcgccctgaagggcgagatcaagcagaggctgaagctgaaggacggcggc<br>cactacgacgctgaggtcaagaccacctacaaggccaagaagcccgtgcagctgcccggcgcctacaacgtcaacatcaagttggaca<br>tcacctcccacaacgaggactacaccatcgtggaacagtacgaacgcgccgagggccgccactccaccggcggcatggacgagctgta<br>caaggtgaagcagaccctgaacttcgatctgctgaagctggccggcgatgtggagagcaaccccgggcccgctagccgactcgagcgg<br>agtactgtcctccgatcggagtactgtcctccgcgaattccggagtactgtcctccgaagacgcgtcgacagtagtgagcaagggcgag<br>gagctgttcaccggggtggtgcccatcctggtcgagctggacggcgacgtaaacggccacaagttcagcgtgtccggcgagggcgagg<br>gcgatgccacctacggcaagctgaccctgaagttcatctgcaccaccggcaagctgcccgtgccctggcccaccctcgtgaccaccctg<br>acctacggcgtgcagtgcttcagccgctaccccgaccacatgaagcagcacgacttcttcaagtccgccatgcccgaaggctacgtcca<br>ggagcgcaccatcttcttcaaggacgacggcaactacaagacccgcgccgaggtgaagttcgagggcgacaccctggtgaaccgcatc<br>gagctgaagggcatcgacttcaaggaggacggcaacatcctggggcacaagctggagtacaactacaacagccacaacgtctatatc<br>atggccgacaagcagaagaacggcatcaaggtgaacttcaagatccgccacaacatcgaggacggcagcgtgcagctcgccgaccac<br>taccagcagaacacccccatcggcgacggccccgtgctgctgcccgacaaccactacctgagcacccagtccgccctgagcaaagacc<br>ccaacgagaagcgcgatcacatggtcctgctggagttcgtgaccgccgccgggatcactctcggcatggacgagctgtacaagtagtga<br>agcagaccctgaacttcgatctgctgaagctggccggcgatgtggagagcaaccccgggcccatggttagtaaaggcgaggagctcttc<br>accggggtcgtgcctatactggtcgaacttgacggtgacgtgaacggacacaaattctctgtgagcggagaaggagagggtgatgcca<br>cttacggaaaactgaccctgaagtttatatgtaccaccggaaagttgcccgttccatggcccactctcgtgacgaccctgacctatggggt<br>ccaatgcttcagccgctatcccgatcatatgaaacaacacgacttcttcaaaagtgccatgccagagggatatgtgcaggagcggacca<br>tcttctttaaagacgatggtaattacaaaacgcgagcagaagttaagtttgagggggatacgttggtgaataggatagaacttaaaggt<br>attgattttaaagaagatggcaatattttgggacacaagctggagtacaattacaattctcacaatgtttatatcatggcggacaaacaa<br>aagaatggcattaaggtgaactttaagattagacataatatcgaggacggaagcgtccaacttgccgaccactatcaacagaatacgc<br>ccatcggcgacggtcctgtcctcttgccggacaaccattacctgtccactcagtccgcattgtccaaagatccaaatgagaaaagagatc<br>atatggtcctgctggagttcgtcactgcggcgggcataacactcgggatggatgagctttataaataa

Legend: mCherry F2A domain Linker sgRNA PAM site sgRNA targeting sequence Stop codon eGFP1 eGFP2

Supplementary Table 2: ssDNA oligonucleotides for cloning

Target	Orientation	Sequence
Targeting sgRNA	Sense	5’-CACCGGGACAGTACTCCGCTCGAGT-3’
	Antisense	5’-AAACACTCGAGCGGAGTACTGTCCC-3’
Non-targeting sgRNA	Sense	5’-CACCGGTCTCTATCACTGATAGGGAG-3’
	Antisense	5’-AAACCTCCCTATCAGTGATAGAGACC-3’

Supplementary Table 3: sgRNAs

Targeting sgRNA	ggacagtactccgctcgagtgttttagagctagaaatagcaagttaaaataaggctagtccgttatcaacttgaaaaagtggcaccgag<br>tcggtgcttttttt
Non-targeting sgRNA	tctctatcactgatagggaggttttagagctagaaatagcaagttaaaataaggctagtccgttatcaacttgaaaaagtggcaccgagt<br>cggtgcttttttt
Pol II-compatible sgRNA	ctgtccctgatgagtccgtgaggacgaaacgagtaagctcgtcggacagtactccgctcgagtgttttagagctagaaatagcaagtta<br>aaataaggctagtccgttatcaacttgaaaaagtggcaccgagtcggtgcttttggccggcatggtcccagcctcctcgctggcgccggc<br>tgggcaacatgcttcggcatggcgaatgggac

Legend: sgRNA targeting sequence Cas9 handle S. Pyogenes terminator Hammerhead ribozyme Hepatitis delta virus ribozyme

Supplementary Table 4: Previously confirmed siRNA sequences

Target	Orientation	Sequence
Cav1	Sense	5′-CCUUCACUGUGACGAAAUACUGGtt-3′
	Antisense	5′-AACCAGUAUUUCGUCACAGUGAAGGUG-3′
CDC42	Sense	5′-CCACAAACAGAUGUAUUUCUAGUct-3′
	Antisense	5′-AGACUAGAAAUACAUCUGUUUGUGGAU-3′
Flot-1	Sense	5′-GGUGAAUCACAAGCCUUUGAGAAca-3′
	Antisense	5′-UGUUCUCAAAGGCUUGUGAUUCACCUG-3′
Rac1	Sense	5′-GGAACUAAACUUGAUCUUAGGGAtg-3′
	Antisense	5′-CAUCCCUAAGAUCAAGUUUAGUUCCCA-3′
RhoA	Sense	5′-CCCAGAUACCGAUGUUAUACUGAtg-3′
	Antisense	5′-CAUCAGUAUAACAUCGGUAUCUGGGUA-3′

siRNAs were previously verified[2]. siRNAs had a single 2-base 3′-overhang on the antisense strand and a blunt end modified with DNA bases (shown in lower case).

Supplementary Table 5: Commercial siRNAs

Target	Product type	Product nr.	Company
Non Coding	siRNA	51-01-14-04	Integrated DNA Technologies
ABL1	ON-TARGETplus Smartpool	L-003100-00-0005	Horizon Discovery LTD
Alix	ON-TARGETplus Smartpool	L-004233-00-0005	Horizon Discovery LTD
ANKFY1	ON-TARGETplus Smartpool	L-013161-00-0005	Horizon Discovery LTD
ARRDC1	ON-TARGETplus Smartpool	L-015918-02-0005	Horizon Discovery LTD
DIAPH1	ON-TARGETplus Smartpool	L-010347-00-0005	Horizon Discovery LTD
ITGB1	ON-TARGETplus Smartpool	L-004506-00-0005	Horizon Discovery LTD
PAK1	ON-TARGETplus Smartpool	L-003521-00-0005	Horizon Discovery LTD
Rab4	ON-TARGETplus Smartpool	L-008539-00-0005	Horizon Discovery LTD
Rab5	ON-TARGETplus Smartpool	L-004009-00-0005	Horizon Discovery LTD
Rab7	ON-TARGETplus Smartpool	L-010388-00-0005	Horizon Discovery LTD
Rab11	ON-TARGETplus Smartpool	L-004726-00-0005	Horizon Discovery LTD
Rab27a	ON-TARGETplus Smartpool	L-004667-00-0005	Horizon Discovery LTD
ROCK1	ON-TARGETplus Smartpool	L-003536-00-0005	Horizon Discovery LTD
Tiam1	ON-TARGETplus Smartpool	L-003932-00-0005	Horizon Discovery LTD
VAV2	ON-TARGETplus Smartpool	L-005199-00-0005	Horizon Discovery LTD

Supplementary Table 6: qPCR primers

Target	Orientation	Sequence
ABL1	Forward	5’-TGAAAAGCTCCGGGTCTTAGG-3’
	Reverse	5’-TTGACTGGCGTGATGTAGTTG-3’
Alix	Forward	5’-ATCGCTGCTAAACATTACCAGTT-3’
	Reverse	5’-AGGGTCCCAACAGTATCTGGA-3’
ANKFY1	Forward	5’-CCATCGTGGCAGACCTCTAC-3’
	Reverse	5’-AGTGGAAGACAAGTTAGCCAGA-3’
ARRDC1	Forward	5’-TAGTGGAGGAGGGTTACTTCAAC-3’
	Reverse	5’-TCTGGGATGCTGTTCAGGTTC-3’
Cav1	Forward	5’-CATCCCGATGGCACTCATCTG-3’
	Reverse	5’-TGCACTGAATCTCAATCAGGAAG-3’
DIAPH1	Forward	5’-CAGTTGGGTGCAAACATTTGG-3’
	Reverse	5’-TCCGGCTATCGTAACTCCCAG-3’
CDC42	Forward	5’-CCATCGGAATATGTACCGACTG-3’
	Reverse	5’-CTCAGCGGTCGTAATCTGTCA-3’
Flot-1	Forward	5’-GCCCTGCATCCAACAGATCC-3’
	Reverse	5’-AATGCCAGTGACTGAGATGGG-3’
GAPDH	Forward	5’-ACAGTCAGCCGCATCTTC-3’
	Reverse	5’-GCCCAATACGACCAAATCC-3’
ITGB1	Forward	5’- CCTACTTCTGCACGATGTGATG-3’
	Reverse	5’- CCTTTGCTACGGTTGGTTACATT-3’
PAK1	Forward	5’-AGGGGAGTTTACGGGAATGC-3’
	Reverse	5’-TCTTCTGCTCCGACTTAGTGATA-3’
Rab27a	Forward	5’-GCTTTGGGAGACTCTGGTGTA-3’
	Reverse	5’-TCAATGCCCACTGTTGTGATAAA-3’
Rab4	Forward	5’- GTCCGTGACGAGAAGTTATTACC-3’
	Reverse	5’- TGAGCGCACTTGTTTCCAAAA-3’
Rab5	Forward	5’- CAAGGCCGACCTAGCAAATAA-3’
	Reverse	5’- GATGTTTTAGCGGATGTCTCCAT-3’
Rab7	Forward	5’- TACAAAGCCACAATAGGAGCTG-3’
	Reverse	5’- GCAGTCTGCACCTCTGTAGAAG-3’
Rab11	Forward	5’- CAACAAGAAGCATCCAGGTTGA-3’
	Reverse	5’- GCACCTACAGCTCCACGATAAT-3’
Rac1	Forward	5’-ATGTCCGTGCAAAGTGGTATC-3’
	Reverse	5’-CTCGGATCGCTTCGTCAAACA-3’
RhoA	Forward	5’-GGAAAGCAGGTAGAGTTGGCT-3’
	Reverse	5’-GGCTGTCGATGGAAAAACACAT-3’
ROCK1	Forward	5’- AACATGCTGCTGGATAAATCTGG-3’
	Reverse	5’- TGTATCACATCGTACCATGCCT-3’
Tiam1	Forward	5’- GATCCACAGGAACTCCGAAGT-3’
	Reverse	5’- GCTCCCGAAGTCTTCTAGGGT-3’
VAV2	Forward	5’- CTGTTTGACCCCTTTGACCTC-3’
	Reverse	5’- GACGCAGTCGTAGATGTCCTC-3’
Targeting sgRNA	Forward	5’-CAGTACTCCGCTCGAGTGTT-3’
	Reverse	5’-GACTCGGTGCCACTTTTTCAA-3’

Supplementary References

1. Shen, M. W. et al. Predictable and precise template-free CRISPR editing of pathogenic variants. Nature **563**, 646–651 (2018) DOI: 10.1038/s41586-018-0686-x.
2. Costa Verdera, H., Gitz-Francois, J. J., Schiffelers, R. M. & Vader, P. Cellular uptake of extracellular vesicles is mediated by clathrin-independent endocytosis and macropinocytosis. *J. Control. Release* **266**, 100–108 (2017) DOI: 10.1016/j.jconrel.2017.09.019.
