# Supplementary material for: A CRISPR-Cas9-based reporter system for single-cell detection of extracellular vesicle-mediated functional transfer of RNA
Source: Nat Commun. 2020 Feb 28;11:1113. doi: 10.1038/s41467-020-14977-8 (PMC7048928; doi:10.1038/s41467-020-14977-8)
Supplement: Supplementary file 2 — Reporting Summary [file 41467_2020_14977_MOESM2_ESM.pdf]

nature research

Corresponding author(s): Dr. Pieter Vader

Last updated by author(s): Feb 5, 2020

Reporting Summary

Nature Research wishes to improve the reproducibility of the work that we publish. This form provides structure for consistency and transparency in reporting. For further information on Nature Research policies, see Authors & Referees and the Editorial Policy Checklist.

Statistics

For all statistical analyses, confirm that the following items are present in the figure legend, table legend, main text, or Methods section.

n/a	Confirmed	
☐	☒	The exact sample size (*n*) for each experimental group/condition, given as a discrete number and unit of measurement
☐	☒	A statement on whether measurements were taken from distinct samples or whether the same sample was measured repeatedly
☐	☒	The statistical test(s) used AND whether they are one- or two-sided<br>*Only common tests should be described solely by name; describe more complex techniques in the Methods section.*
☒	☐	A description of all covariates tested
☐	☒	A description of any assumptions or corrections, such as tests of normality and adjustment for multiple comparisons
☐	☒	A full description of the statistical parameters including central tendency (e.g. means) or other basic estimates (e.g. regression coefficient) AND variation (e.g. standard deviation) or associated estimates of uncertainty (e.g. confidence intervals)
☐	☒	For null hypothesis testing, the test statistic (e.g. *F*, *t*, *r*) with confidence intervals, effect sizes, degrees of freedom and *P* value noted<br>*Give P values as exact values whenever suitable.*
☒	☐	For Bayesian analysis, information on the choice of priors and Markov chain Monte Carlo settings
☒	☐	For hierarchical and complex designs, identification of the appropriate level for tests and full reporting of outcomes
☒	☐	Estimates of effect sizes (e.g. Cohen's *d*, Pearson's *r*), indicating how they were calculated

Our web collection on statistics for biologists contains articles on many of the points above.

Software and code

Policy information about availability of computer code

Data collection	Data was collected using up-to-date software provided by the manufacturer for all the equipment listed in the Methods section.
Data analysis	Fluorescence and confocal microscopy were processed using ImageJ 1.47v, flow cytometry data was analyzed using FlowJo v10, statistical analyses were performed using Graphpad Prism 8.0.1. In silico analysis of confocal pictures was performed using Columbus software v2.7.1 (Perkin-Elmer). Nanoparticle tracking analysis measurements were analyzed using NTA software v3.1.

For manuscripts utilizing custom algorithms or software that are central to the research but not yet described in published literature, software must be made available to editors/reviewers. We strongly encourage code deposition in a community repository (e.g. GitHub). See the Nature Research guidelines for submitting code & software for further information.

Data

Policy information about availability of data

All manuscripts must include a data availability statement. This statement should provide the following information, where applicable:
- Accession codes, unique identifiers, or web links for publicly available datasets
- A list of figures that have associated raw data
- A description of any restrictions on data availability

The datasets generated during and/or analysed during the current study are available from the corresponding author on reasonable request.

Field-specific reporting

Please select the one below that is the best fit for your research. If you are not sure, read the appropriate sections before making your selection.

☒ Life sciences ☐ Behavioural & social sciences ☐ Ecological, evolutionary & environmental sciences

For a reference copy of the document with all sections, see nature.com/documents/nr-reporting-summary-flat.pdf

Life sciences study design

All studies must disclose on these points even when the disclosure is negative.

Sample size

No sample size-calculation was performed prior to experiments, as no preliminary data was available on effect size and variation. Experiments were performed 3 - 4 times, and analyzed using the statistical analyses listed throughout the manuscript. In no case were 1-sided statistical tests performed. For flow cytometry, at least 50.000 events/sample (and generally 100.000 events/sample) were analyzed to ensure sufficient resolution and accuracy for measurement and analysis.

Data exclusions

Some siRNAs were excluded based on the following pre-established exclusion criteria: 1: Insufficient knockdown 2: High levels of toxicity.

Replication

Experiments were performed successfully at least 3 times. Furthermore, key experiments were validated in additional cell lines.

Randomization

Samples were not randomized during this study. It is unlikely that this has affected results, as all samples were measured at the same time using the same machine settings (eg. laser voltage, camera settings, etc...). Moreover, all samples were analyzed using the exact same gates, analytical formulas and settings.

Blinding

Samples were not blinded as all samples were measured at the same time using the same machine settings (eg. laser voltage, camera settings, etc...). Moreover, all samples were analyzed using the exact same gates, analytical formulas and settings.

Reporting for specific materials, systems and methods

We require information from authors about some types of materials, experimental systems and methods used in many studies. Here, indicate whether each material, system or method listed is relevant to your study. If you are not sure if a list item applies to your research, read the appropriate section before selecting a response.

Materials & experimental systems

n/a	Involved in the study	
☐	☒	Antibodies
☐	☒	Eukaryotic cell lines
☒	☐	Palaeontology
☒	☐	Animals and other organisms
☒	☐	Human research participants
☒	☐	Clinical data

Methods

n/a	Involved in the study	
☒	☐	ChIP-seq
☐	☒	Flow cytometry
☒	☐	MRI-based neuroimaging

Antibodies

Antibodies used

As described in the Methods section:
Membranes were subsequently probed using the following antibodies: Alix 1:1000 (Thermo Fisher Scientific, MA1-83977), Calnexin 1:1000 (GeneTex, GTX101676), CD9 1:1000 (Abcam, ab92726), CD63 1:1000 (AB8219), Flot-1 1:1000 (Cell Signaling Technology, 3253), TSG101 1:1000 (Abcam, ab30871) and H2B 1:1000 (Abcam, ab52599) in staining buffer consisting of 1 part Odyssey Blocking Buffer and 1 part TBS with 0.1% Tween-20 (TBST). Secondary antibodies consisted of either anti-rabbit IgG conjugated to AlexaFluor 680 (Thermo Fisher Scientific, A-21076) or anti-mouse IgG conjugated to IRDye 800CW and were applied at a 1:10,000 dilution in staining buffer. Proteins were visualized using an Odyssey Infrared Imager (LI-COR Biosciences) at 700 and 800 nm.

Validation

Alix MA1-83977 was verified by Sigma-Aldrich by probing Alix WT and knockdown samples.
Calnexin GTX101676 was verified by GeneTex by probing Calnexin WT and knockdown samples.
CD9 ab92726 was verified by Abcam by probing CD9 WT and KO samples.
CD63 AB8219 was not verified using KD/KO by Abcam, but Abcam lists its use in 63 publications, shows a band at the expected height, and shows enrichment in EVs as compared to cell lysate, as is expected for CD63.
Flot-1 3253 was not verified using KD/KO by Cell Signalling but is listed by CiteAb in 15 publications, shows a band at the expected height, and shows enrichment in EVs as compared to cell lysate, as is expected for Flot-1.
TSG-101 ab30871 was not verified using KD/KO by Abcam, but Abcam lists its use in 45 publications, shows a band at the expected height, and shows enrichment in EVs as compared to cell lysate, and shows immunohistochemistry in HeLa cells were its compartmentalized localization is in line with scientific literature on TSG101.

H2B ab52599 as not verified using KD/KO by Abcam, but Abcam lists its use in 11 publications, shows a band at the expected height, and shows nuclear localization using immunohistochemistry, as is in line with scientific literature on H2B.

Eukaryotic cell lines

Policy information about cell lines

Cell line source(s)	T47D cells were ordered from Sigma-Aldrich.<br>HEK293T and HeLa cells were ordered from the ATCC.<br>MDA-MB-231 cells were kindly provided by dr. SJ Vervoort and Prof. dr. P. Coffer from the UMC Utrecht (as used in: eLife, 2018, Global transcriptional analysis identifies a novel role for SOX4 in tumor-induced angiogenesis.). Original source: ATCC.<br>HMEC-1 cells were kindly provided by dr. Ades from the Centers for Disease Control and prevention, Atlanta, GA (as described in: J. Invest. Dermatol., 1992, HMEC-1: establishment of an immortalized human microvascular endothelial cell line by Ades et al.). Original source: ATCC.<br>MCF-7 cells were kindly provided by dr. Anoek Zomer and prof. dr. J. van Rheenen from the Hubrecht Institute, Utrecht, The Netherlands (as used in: Cell, 2015, In Vivo Imaging Reveals Extracellular Vesicle-Mediated Phenocopying of Metastatic Behavior). Original source: ATCC.
Authentication	Cell lines were authenticated by morphological assessment through light microscopy.
Mycoplasma contamination	All cell lines were tested negative for mycoplasma.
Commonly misidentified lines<br>(See ICLAC register)	No commonly misidentified lines were used in this study.

Flow Cytometry

Plots

Confirm that:

- [x] The axis labels state the marker and fluorochrome used (e.g. CD4-FITC).
- [x] The axis scales are clearly visible. Include numbers along axes only for bottom left plot of group (a 'group' is an analysis of identical markers).
- [x] All plots are contour plots with outliers or pseudocolor plots.
- [x] A numerical value for number of cells or percentage (with statistics) is provided.

Methodology

Sample preparation	As described in the Methods section: "For flow cytometry analysis cells were trypsinized for 5 minutes using TrypLE Express (Thermo Fisher Scientific), and transferred to 5 ml flow cytometry tubes using a similar volume of DMEM containing 10% FBS. Cells were centrifuged for 5 minutes at 300 x g, washed in 5 ml 1% FBS in PBS, and centrifuged once more for 5 minutes at 300 x g. Cells were then resuspended in 250 µl 1% FBS in PBS, and kept on ice until flow further analysis."
Instrument	As described in the Methods section: "Cells were analyzed on an ImageStream Mark II (Amnis), MacsQuant VYB (Miltenyi Biotec), or Fortessa (BD Biosciences) flow cytometer (...)"
Software	As described in the Methods section: "(...) and further analyzed using FlowJo v10 software. "
Cell population abundance	For flow cytometry, at least 50.000 events/sample (and generally 100.000 events/sample) were analyzed to ensure sufficient resolution and accuracy for measurement and analysis.
Gating strategy	For analyses performed with MacsQuant and Fortessa flow cytometers:<br>Firstly, cell debris was gated out and cells were selected using FSC and SSC.<br>Then, single cells were selected by plotting SSC-A vs SSC-H.<br>Where applicable, viable cells were selected using DAPI staining (FSC-UV plot, high signal excluded).<br>Then, donor and reporter cell populations (Stoplight- vs Stoplight+) were selected by mCherry signal using the Yellow-Green laser (FSC-YeGr plot).<br>For the reporter cells (mCherry+ gated), reporter activation was assessed by measuring eGFP expression using a blue (488 nm) laser (FSC-Blue plot). eGFP+ cells were counted as activated reporter cells.<br><br>For analysis performed with the Imagestream Mark II:<br>Only the SSC, 488 and 561 lasers were activated for analysis<br>Firstly cell debris and calibration beads were gated out, and single cells were selected by plotting the Area and Aspect Ratio in Channel 1 (brightfield).<br>Then, donor and reporter cell populations (Stoplight- vs Stoplight+) were selected by mCherry signal using the 561 laser in Channel 4 (595-640 band) (Area_01 vs Intensity_Ch04 plot).<br>For the reporter cells (mCherry+ gated), reporter activation was assessed by measuring eGFP expression using the 488 laser in

Channel 2 (480-560 band) Area_01 vs Intensity_Ch02 plot). eGFP+ cells were counted as activated reporter cells.

For all analyses: both compensation matrixes and gating settings were set based on signals in all measured channels using donor cells (mCherry-eGFP-), untreated reporter cells (mCherry+eGFP-) and both targeting and non-targeting sgRNA transfected reporter cells (mCherry+eGFP- and mCherry+eGFP+ respectively). As different cell types showed different expression levels of the reporter constructs, these matrixes and gates were set up separately for each cell type. However, the same gates and matrixes were used for all samples and conditions within the same experiment per cell type.

☒ Tick this box to confirm that a figure exemplifying the gating strategy is provided in the Supplementary Information.
